# Electrochemical Sensors and Biosensors for the Determination of Food Nutritional and Bioactive Compounds: Recent Advances

**DOI:** 10.3390/s24206588

**Published:** 2024-10-12

**Authors:** Barbara Brunetti

**Affiliations:** Department of Food, Environmental and Nutritional Sciences (DeFENS)DeFENS, University of Milan, Via Celoria 2, I-20133 Milan, Italy; barbara.brunetti@unimi.it; Tel.: +39-02-50319231

**Keywords:** electroanalysis, electrodes, biosensors, food analysis, nutrients

## Abstract

The significance of food nutrients and bioactive compounds in human health has driven the development of many methods for their determination in different matrices. Among these, electroanalysis has gained popularity due to its cost-effectiveness, rapidity, and, in many cases, portability and minimal sample treatment. This review highlights key advances in electrochemical sensors and biosensors from 2019 to the present. Given the variability and the challenges of managing food matrices, the focus is limited to methods that have been thoroughly assessed for their applicability to real samples. The technical characteristics and analytical performance of the proposed sensors are discussed, along with breakthrough features and future trends.

## 1. Introduction

The term “nutrient” defines compounds needed for the effective functioning of the human organism [1]. The main classes are represented by carbohydrates, proteins, fats, vitamins, and minerals. Macronutrients (carbohydrates, proteins, and fats) are the major sources of energy and the building materials for living cells. Micronutrients, such as vitamins and minerals, are needed in smaller amounts but are still essential. Many nutrients also have a synergetic effect: they support each other in promoting health. In addition to nutrients, phytochemicals such as polyphenols have also been found to be essential thanks to their many health-promoting functions. Their significant role in nutrition is well documented [2].

Some of these compounds are also intentionally added to foodstuffs to increase their nutritional value. Today, fortified and functional foods are widely available and popular among consumers. On the other hand, the demand for low-fat, low-sugar products has prompted changes in the production process to decrease the level of these nutrients.

For all these reasons, the quantification of nutritional and bioactive species is of great interest in any kind of food matrix, from raw products to processed goods. However, there are some challenges to be addressed. Food matrices require versatile methods since they vary greatly in composition and complexity, from water-rich vegetables and fruit to heterogeneous dairy products and meats, lipophilic matrices as oils and solid fats, and so on. Moreover, the simultaneous quantification of several compounds could be required. Rapidity and high throughput sampling could also be needed, especially for monitoring purposes in the production steps and during storage. Furthermore, there is a constantly increasing demand for easy, fast, and cheap methods and miniaturized instruments for decentralized testing in small control labs and even in the household.

Nowadays, food analysis is mainly led by chromatographic methods, owing to their ability to provide information about multiple analytes. However, they are affected by the low speed of analysis, bulky equipment, high maintenance costs, and the need for trained personnel. Spectrophotometric methods are also widely used and popular, but they still face some limitations, including spectral interferences from matrix components or coexisting substances, low sensitivity, and laborious sample pretreatment. An advantageous alternative is offered by electroanalysis, which is attracting increasing attention because of its outstanding performance. As is well known, electrochemical methods are commonly characterized by lower cost, simple use, and higher speed of analysis. Despite these benefits, conventional electrode materials like glassy carbon and metals are prone to fouling and difficult to clean. They also suffer from limited selectivity. However, innovative approaches, such as screen printing technology, nanostructured and porous materials, protective and molecularly imprinted polymers, enzymes, and other biological materials, can improve the selectivity, prevent unwanted adsorption phenomena, and enhance the sensitivity toward various species.

These features have led electroanalysis to be a promising front-runner also in the field of food analysis. Moreover, the portable character of the most recent instrumentation, coupled with the availability of disposable parts, holds considerable potential for decentralized testing, especially in locations where sophisticated instrumentation is not practical and/or available.

This article reviews the most significant advances relevant to the electrochemical determination of food nutrients and other bioactive compounds from 2019 to the present. Since the attention is focused on the above-mentioned advantages, only stand-alone electrochemical devices are covered (not detectors for chromatography and/or electrophoresis). Papers were selected based on their analytical characterization, including evaluation of common analytical parameters such as linear range, limit of detection, repeatability/reproducibility, and selectivity. Only papers demonstrating applicability to real food samples and evaluating accuracy through comparison with reference methods or known values were cited. Actually, accuracy is often evaluated solely through recovery experiments, but this approach is controversial since a good recovery could suggest the absence of major bias but does not guarantee good trueness [3]. The main weakness of this test is that the added analyte may not be in the same state as the one originally present in the sample.

Moreover, papers reporting two or more linear ranges were also left out, as this suggests that the functional relationship is not linear. In the author’s opinion, the range should be shortened, or a different type of regression should be used [3].

## 2. Nutrients

Several nutrients are inherently electroactive, allowing for their direct investigation through electrochemical methods. Among them, it is worth mentioning the vitamins, both water- and fat-soluble, and many amino acids such as phenylalanine, tryptophan, and L- tyrosine. Essential amino acids are significant in food analysis not only from a nutritional point of view but also because they contribute to the aroma and taste of the product. For these reasons, they are extensively studied in several different food matrices. Cholesterol is a very interesting case. Its redox activity was discovered relatively late [4]. Its oxidation potential is very high at common electrode surfaces, and this is detrimental in food matrices because of the presence of many other electroactive compounds. Even for sugars (mainly glucose and fructose), whose determination is traditionally the prerogative of biosensors, many non-enzymatic methods were developed.

Interestingly, electrochemistry in organic solvents has also been extensively explored, leading to novel methods for determining lipophilic species.

### 2.1. Electrochemical Sensors for Determining Nutrients

Table 1 reports the latest electrochemical sensors for nutrient determination, along with the main technical features and some key parameters such as linear range and limit of detection (LOD).

As can be seen, several chemically modified electrodes for a large variety of foods were recently proposed [5,6,7,8,9,10,11,12,13,14,15,16,17,18,19,20,21,22,23,24,25,26,27,28,29,30,31,32,33,34,35,36,37,38,39,40,41,42,43,44,45,46,47,48,49,50,51].

The most common electrode substrate is glassy carbon (GC). This material is characterized by favorable features such as good current conductivity, high stability in any media, sturdiness, easy cleaning, and low cost. In all the reported cases, the GC surface is modified by nanostructured materials, especially carbon nanotubes (CNTs) [34,37,44,49] and graphene (GR) species such as GR oxide (GO), reduced GO (RGO), and electrochemically RGO (ERGO) [5,9,31,36,50]. Nanomaterials are a very valuable option because they could increase the electroactive surface area and enhance electron transfer. Moreover, adsorption of the targeted species could be facilitated.

Duzmen et al. [34] introduced a novel nanocomposite made of carbon nanotubes (CNTs), poly(aminopyrazine), and ZrO_2_ nanoparticles (NPs). A CNTs and ZrO_2_ NPs solution was drop-casted onto the base GC electrode, then the modified electrode was immersed in an aminopyrazine solution for electropolymerization by cyclic voltammetry. The resulting sensor was used for determining ascorbic acid in fruit juice. It exhibited higher sensitivity compared to the unmodified electrode, along with good selectivity and electrocatalytic activity toward the analyte.

Another composite CNT material was proposed by Vaschetti et al. [37]. In this work, a hybrid structure made of a mesoporous material synthesized by the authors and CNTs was prepared using an experimental design based on Central Composite Design/Response Surface Methodology. Through this method, CNTs/mesoporous material concentration and sonication time were optimized in several solvents. The selected hybrid, drop-casted onto a GC electrode, produced a valuable sensor for the selective determination of ascorbic acid in several food and related samples.

Very recently, a nanocomposite made of carbon nanotubes functionalized with cobalt nanoparticles was used as a modifier for determining folic acid [44]. Results obtained analyzing fruit juices were in accordance with those of a reference HPLC method. The sensor allowed also the determination of quercetin in plant extracts, but simultaneous quantification of the two species was not carried out in real samples.

Avan et al. [49] demonstrated that the functionalization of β-cyclodextrin on CNTs increases their chemical suitability and dissolution attributes and, therefore, their electrochemical reactivity. In this work, the composite nanostructured material was obtained by dispersing CNTs in a β-cyclodextrin solution for some hours. The resulting suspension was then drop-casted onto the GC electrode. The obtained sensor exhibited excellent features in the simultaneous determination of fat-soluble vitamins. Well-defined and separated peaks relevant to vitamins A, E, D_3_, and K_1_ in both cyclic and square wave voltammograms were detected. The proposed approach was successfully applied to the analysis of milk, as well as pharmaceutical samples.

Salemi et al. [5] described the ability of a GC electrode modified with a MnO_2_/N-doped reduced graphene oxide (RGO) nanocomposite to determine phenylalanine. The improvement of the electron transfer capability and redox reversibility was demonstrated by comparing the behavior of the modified and unmodified electrodes in a K_4_[Fe(CN)_6_] test solution. Moreover, EIS experiments demonstrated a lower resistance in the modified sensor. In the analyte solution, it showed a well-defined peak, while the base GC electrode did not show any signal. The proposed procedure was applied to the analysis of food and drug samples with results comparable to a standard HPLC method.

Ahmadi Direstani et al. [9] reported a nanocomposite made of P- doped ERGO, 1,4-bis(triphenylphosphonium)butane (BTPB), and SiW_11_O_39_Ni(H_2_O) (SiW_11_Ni), which was formed onto a GC surface. The hybrid material was synthesized via an electrodeposition procedure. Firstly, a (BTPB)SiW_11_Ni/GO suspension was drop-casted onto the GC electrode, then the electrode was immersed in a solution containing H_3_PO_4_ and BTPB. P-doped ERGO was formed on the surface of the GC electrode by electrochemical reduction using chronoamperometry. Surface morphology, electrochemical, and electrocatalysis properties were studied by several techniques. The obtained sensor exhibited good sensing performance for L-tryptophan in milk and egg white. L-cysteine was also tested, but it was not found in the selected samples.

De Faria et al. [36] developed a simple and sensitive method using flow injection analysis (FIA) with amperometric detection using an RGO-modified GC electrode. As frequently seen, the electrode modification was obtained by drop-casting an RGO suspension (in dimethylformamide). Several milk-based beverages were tested, obtaining results comparable to a standard chromatographic method.

Gold nanostructures were also exploited in conjunction with a carbon nanomaterial to enhance the electrocatalytic properties [31]. Herein, the sensor obtained by drop-casting a gold nanocages/fluorinated GR nanocomposite on a GC electrode was used for determining selenium in food (and environmental) samples. Gold nanocages were synthesized via a one-pot method without the use of any solid templates or Au seeds, while GR was fluorinated directly using F_2_. The two solutions were mixed together with a Nafion dispersion (in ethanol). The role of the polymer was to increase the viscosity of the dispersion to prevent it from falling off during the drop-casting.

Similar gold nanostructures, i.e., hierarchical dendritic ones, were used for obtaining a sensor for the same analyte [32]. These nanoparticles, characterized by a unique shape, are hyperbranched structures with high surface area and electrochemical activity. Modification of the GC electrode was achieved by potentiostatic electrodeposition in a HAuCl_4_ solution using Na_2_SO_4_ as a supporting electrolyte.

Metal oxide nanoparticles were also employed [12,17,21]. A unique nanomaterial made of cuprous oxide micro-nano cubes was prepared as a modifier for a GC electrode [17]. Cu_2_O micro-nano cubes were synthesized via a seed-medium process. The formation mechanism is detailed by the authors in three steps: nucleation, followed by the crystal nucleus growth and, lastly, by the stacking/merging growth. The obtained sensor was successfully applied to the enzyme-free determination of glucose in some beverages.

Ni oxide nanoparticles were obtained from a precursor powder of Ni(OH)_2_ calcinated at a very high temperature [21]. The obtained powder was dispersed in water and then drop-casted onto a GC electrode. The obtained sensor was used for total glucose determination in commercial beverages.

A nanocomposite made of zinc oxide nanoparticles and a molecularly imprinted polymer was used for enhancing a GCE’s selectivity and sensitivity toward tyrosine [12]. The determination of the analyte was carried out not only in a food sample (egg) but also in human fluids. The results were comparable to those obtained with a tyrosine assay kit.

Porada et al. [46] proposed a zeolite composite as a modifier. Zeolite could be a valid alternative to more commonly known materials due to its molecular sieve properties, ability to incorporate transition metals for increasing catalytic reactivity, thermal and chemical stability, insolubility in most organic solvents, resistance in acid media, nontoxicity, high surface area, availability in nature, and lower cost [52]. In this work, the authors propose a Ni-zeolite/carbon black hybrid nanocomposite-modified GC for determining some B-group vitamins in several foods. Interestingly, vitamins B_2_, B_9_, B_12_, and B_3_ were detected simultaneously without any signal overlapping, and detection limits were in the nanomolar range.

A multivitamin sensor was obtained by modifying a GC electrode with nitrogen and sulfur co-doped graphene quantum dots (GQDs) immobilized in chitosan [50]. Doping with heteroatoms generates permanent dipoles in the GR structure, providing increased surface reactivity and catalytic activity. GQDs were synthesized by a simple and fast pyrolysis method. The obtained sensor was used for determining vitamins B_12_, B_2_, and B_6_ in beverages. Results were in accordance with the values reported on the product’s label.

Many screen-printed sensors were proposed [6,8,10,13,20,22,27,35,48,51]. Screen printing is a very versatile technology that allows the design of personalized electrode surfaces and their tailored modification [53]. As recently pointed out [53], SPEs could be a valuable answer to GAC (Green Analytical Chemistry) requirements since they offer the opportunity to use lower sample volumes and avoid the cleaning step, which is both time- and material-consuming. Moreover, these platforms could be easily coupled with portable instruments, favoring decentralized testing.

Wong et al. [6] reported the development of an SPE platform made of flexible polyester sheets modified with a nanocomposite material. The sensor was applied to the determination of tryptophan in food samples such as milk and dark chocolate. Comparison of the obtained results with those from a standard HPLC method and the evaluation of possible interferents confirms the suitability of the sensor for food analysis.

**Figure 1:**
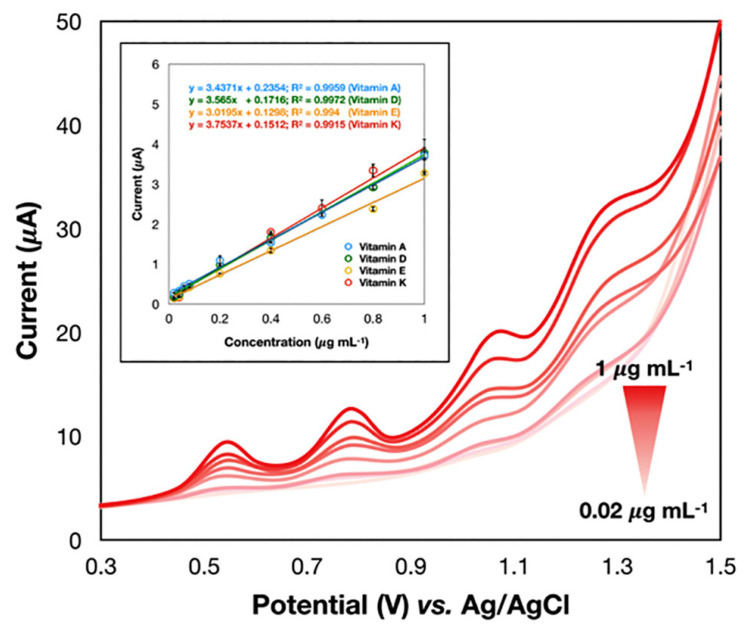
Voltammograms recorded at the newly proposed GR–Nafion/SPE sensor. Electrochemical oxidation of vitamins A, D, E, and K and the representative calibration graph (inset) showing the anodic peak current and the concentration of the vitamins. Reproduced with permission from [48].

Thangphatthanarungruang et al. [48] described a screen-printed graphene nanocomposite with the polymer Nafion, known for its appealing antifouling properties. Fat-soluble vitamins (A, D, E, and K) are determined in diverse food samples (milk, yogurt, and parsley) using adsorptive square wave voltammetry. Remarkably, this proposal addresses two major challenges of electroanalysis: simultaneous determination of several analytes and analysis of fat-soluble species. As can be seen in Figure 1, well-separated oxidation peaks can be detected and accurately measured. Moreover, the graphene/Nafion nanocomposite SPE enhances the signals compared to conventional electrodes. The proposed method’s accuracy was assured by comparison with a standard HPLC-UV method. Therefore, it has great potential for the simultaneous determination of fat-soluble vitamins in real food samples.

A screen-printed graphene electrode (SPGE), a well-known conductive material with demonstrated excellent properties, was proposed for the determination of vitamin K_1_ in green vegetables using square wave anodic stripping voltammetry [51]. Results were in good agreement with those from a standard chromatographic method.

Garg et al. [10] described an easy procedure for functionalizing an SPE surface with boron carbon nitride (BCN). A five-fold increase in current response compared to the bare electrode was demonstrated for the quantification of tryptophan. Sensor stability, reproducibility, and accuracy were all tested and they proved the successful ability to detect the analyte in complex food samples such as egg white.

Other proposals include the use of commercial SPEs modified with nanocomposites [8,13,20,22]. A composite of GO and hydroxyapatite (HAp) was proposed as a modifier for a tryptophan sensor [8]. HAp particles were grown on GO sheets via a heterogeneous nucleation process in a simulated body fluid (SBF). The SBF was used as a supply of calcium and phosphate ions for HAp crystallization. The synergistic effect of the porous material with GO appeared to improve the analyte detection. The proposed method was applied to the determination of tryptophan in sunflower and pumpkin seeds with satisfactory results, comparable to those obtained by a classic HPLC method.

A new Schiff base Ni complex has been synthesized and then electropolymerized onto an SPE modified with CNT [13]. The resulting sensor was coupled with chemometric tools for determining monosaccharides. A strong electrocatalytic activity toward the oxidation of glucose and fructose in alkaline media was demonstrated. Partial least squares regression was used to resolve mixtures of glucose and fructose in honey. The new method’s accuracy was assured by statistical comparison with a reference HPLC method.

An SPE modified with a 3D composite material made of carboxylated graphene/carboxylated multi-walled CNTs and gold nanoparticles was applied for the non-enzymatic determination of reducing sugars [20]. The nanocomposite showed good catalysis behavior toward several sugars: glucose, fructose, arabinose, mannose, xylose, and galactose. The proposed method was applied to the determination of glucose and fructose in apple juice with satisfactory results. The authors suggest this analytical system as an in situ sensor since the modified SPE could be inserted directly into a fruit piece and connected to a portable potentiostat.

A composite of citrate-modified β-cyclodextrin (CIT-BCD) and magnetic Fe_3_O_4_ nanoparticles was used for detecting cholesterol at an SPE [22]. Unlike the previously reported papers, the nanocomposite was used in solution, not attached to the electrode surface. Cholesterol was detected on the basis of the competition of the inclusion complex formation with CIT-BCD and cholesterol, and between CIT-BCD and methylene blue, using the magnetic particles as a support medium for BCD. Citrate was used to improve the analyte loading capacity. Amperometry results showed good linearity in a large concentration range. The method was used on a corned beef sample with results comparable with a reference HPLC method.

A commercial SPE platform was connected with a newly developed portable device that is remarkably low-cost and easy to use [35]. It was tested for determining ascorbic acid in fruit juices. The relevant method has several advantages: in-field portability, short analysis time, low cost per determination, adequate detection range, and no sample pretreatment. The obtained values were confirmed by a standard HPLC method.

Some ion-selective electrodes (ISE) were proposed for the potentiometric determination of potassium [25,26,27], iron [27], calcium [24], and phosphorus (as phosphate) [30]. Yoon et al. [25] used screen printing technology to assemble a potentiometric sensor for K^+^. Interestingly, the authors proposed a whole device made by combining the SPE with a printed circuit board. The collected data could be transferred to a mobile application through a Bluetooth module. The accuracy of the measurement in commercial beverages was demonstrated by comparison with an independent electrochemical analyzer.

In a more recent study, another K^+^–selective electrode was developed by combining potentiometry with the unique properties of a porphyrin derivative species [26]. The ionophore was synthesized and incorporated into a PVC membrane. This sensor combines the advantages of potentiometry, such as wide linear range, low detection limit, fast response time, high stability, good selectivity, and repeatability, with the capability of working in a wide pH range, simple design, low cost, and easy fabrication. It is able to detect potassium accurately in many matrices, not only foods.

A very sensitive ISE for phosphate determination was fabricated using Ba_3_PO_4_, Cu_2_S, and Ag_2_S solids [30]. The solids were mixed and compressed to obtain a pellet that was connected to a copper wire fixed to a glass tube. The proposed sensor is easy to prepare and, according to the authors, is lower in cost and with a longer lifetime compared to similar devices. Phosphate was accurately determined as phosphorus in some complex matrices such as beef, beans, garlic, and dried apricots.

Kul et al. [27] developed screen-printed platforms for the potentiometric determination of both K^+^ and Fe^3+^ ions. The SPEs were modified with a carbon black nanomaterial, and the selective membrane included zinc (II) phthalocyanine as the ionophore. Thanks to the use of a portable potentiostat and a smartphone application, the method is a promising candidate for in-field analysis.

Iron was also determined using a novel lab-made sensor made of a borate glassy matrix, doped with molybdenum, lithium, and vanadium oxides, and surface modified with carbon nanoparticles [29]. The nanoparticles were synthesized by means of an environmentally friendly method, using honey instead of toxic materials as the precursor. Also, in this case, using a portable potentiostat, in situ detection could be possible.

Carbon paste electrodes (CPE) were also proposed [7,24,42,47]. As is well known [54], CPEs have important advantages such as surface renewability, easy incorporation of catalysts and related species, high electron transfer rate, large potential windows, and lower ohmic resistance compared to other electrode materials. A CPE made with a silver zeolite nanocomposite was proposed for the determination of tryptophan in milk and wheat flour [7]. Silver is known to strongly bind proteins and peptides, but its nanoparticles are easy to agglomerate. In this work, the problem was overcome by its combination with zeolites. The obtained nanocomposite was mixed with graphite powder and paraffin oil to obtain a homogeneous paste. To assemble the sensor, the paste was pushed into a glass tube, and a copper wire was inserted to establish the electrical connection. The sensor’s analytical features make it a valid alternative to more expensive and complicated tryptophan (bio)sensors.

CPEs made by combining graphite, a plasticizer, and a metal-organic framework structure with/without graphitic carbon nitride were proposed for the potentiometric determination of calcium ions [24]. The sensors were applied to the analysis of milk powder and validated against an HPLC method.

A magnetic porous imprinted polymer (MPIP) was used as the base material for constructing a folate CPE [42]. The MPIP was obtained using a magnetic water-dispersible nanomaterial as a starter. It is worth noting that complex theoretical studies were carried out to select the best functional monomer. A comparison of the analytical features of the CPEs with and without the newly synthesized MPIP confirmed its outstanding ability to selectively detect folate in the micromolar range.

Tesfaye et al. [47] described a poly(glutamic acid)/zinc oxide NPs modified CPE for the determination of riboflavin. The polymerization was carried out at the zinc oxide nanoparticles on CPE immersed in a glutamic acid solution by cyclic voltammetry. The electrochemical properties and analytical features of bare and modified CPEs were accurately evaluated. The newly proposed CPE proved superior in determining riboflavin, demonstrating the active role of the nanocomposite. The large effective surface area and strong accumulation ability produced higher sensitivity and a lower detection limit.

Various other electrode materials were proposed. Ayranci et al. [14] developed a sensor for the detection of glucose based on a graphite rod electrode modified with monodisperse Pt–Ni nanocomposites decorated on RGO. This material was synthesized using a new ultrasonic hydroxide-supported reduction method. The aim of the authors was to enhance the electrocatalytic activity and to avoid the poisoning of the electrode surface during the oxidation of glucose. The sensor was tested in various beverages with satisfactory results. Another graphite-based sensor was proposed for the determination of folic acid in various food extracts [45]. Graphite was imbued with a molecularly imprinted polymer (polyacrylonitrile). The electrode exhibited high reproducibility and good stability even after prolonged usage. A pyrolytic graphite sheet electrode was modified with gold nanoparticles and used for determining ascorbic acid in beverages [38]. The nanomaterial was deposited using a spontaneous adsorption technique, and the obtained sensor demonstrated very satisfactory performance characteristics. The results were confirmed by comparison with a conventional UV-Vis spectrophotometric method.

A carbon fiber electrode was modified by layering a 3D vertical porous array and in situ grown “tentacle” N-doped CNTs [39]. The resulting sensor was successfully applied to the determination of ascorbic acid in beverages. According to the authors, the appealing characteristics were due to the boosted electrocatalytic activity promoted by the innovative nanocomposite material.

A hierarchical composite consisting of NiCo_2_O_4_ nanorods/nanoparticles was used as the working electrode to determine glucose [15]. Transition metal oxides are known and widely used because of their low-cost synthesis, and nickel/cobalt-based nanomaterials in particular have shown good electrocatalytic activity toward glucose. The composite was crystallized on nickel foam. The combination of heterogeneous nanoarrays enhanced the specific surface area, electrical conductivity, and electrocatalytic activity toward glucose compared to the NiCo_2_O_4_ nanorods alone. Glucose in commercial beverages was determined by amperometry. The results were in agreement with those of a commercial glucose meter. Another glucose sensor built on a nickel foam base was developed using NiCo-layered double hydroxide nanosheet arrays [18]. This material showed uniform dispersion without agglomeration, a high electrochemically active surface area, and excellent electron transfer efficiency. As a consequence, the obtained sensor exhibited satisfactory reproducibility and selectivity for glucose determination in many food samples, such as fruits, vegetables, milk, and beverages.

Carbon cloth was used as a support material in the development of a glucose sensor [16]. In this study, Mn-doped NiO nanosheets display excellent catalytic activity. The resulting sensor showed good performance in the determination of glucose in sports beverages.

An innovative composite film-modified electrode based on Dawson polyoxometalates and an ionic liquid-decorated CNT material was constructed for the determination of L-tyrosine [11]. The composite was fabricated by using a layer-by-layer self-assembly method. The combination of the active components offered higher conductivity and increased contact area. A modified ITO electrode exhibited good sensing performance in a wide linear range with high selectivity for common interferences and good stability. The analysis of L-tyrosine in meat floss demonstrated the method’s applicability to complex food matrices. Another modified ITO sensor was proposed by Zhang et al. [33] for the determination of ascorbic acid. In this work, the modifier is made of several components: a polyoxometalate, Pd nanoparticles, and also chitosan to prevent nanoparticle aggregation. The chitosan-Pd matrix carries a partial positive charge that can interact with the negatively charged ascorbate ion. The sensing system was successfully applied to the analysis of ascorbic acid in juice, and the results were comparable with those obtained by standard HPLC and spectrophotometric methods.

An iodine-coated platinum electrode was prepared for the determination of iron using linear sweep voltammetry [28]. Iodine prevents unwanted adsorption and surface processes usually observed at bare Pt electrodes. The method showed adequate sensitivity and selectivity for the determination of iron content in spinach. Comparison with a standard ICP-OES method confirmed the method’s accuracy.

A 3D-printed sensing device was also proposed [43]. All the components of the electrochemical cell were manufactured by a method previously established by the authors. The working electrode was developed using carbon black as the base material. The device was used for determining folic acid in some foods by square wave voltammetry. Interestingly, the ecological profile was also calculated.

Lipophilic species investigation can be troublesome. Several new procedures were implemented to address this challenge. Square wave voltammetry of microdroplets immobilized on a paraffin-impregnated graphite electrode was used to study astaxanthin oxidation [40]. The proposed three-phase method could be a valid alternative to the use of organic solvents as working media. It was optimized for determining the analyte in crustaceans (shrimp and soft-shell crab) and validated by comparison with a spectrophotometric method.

A voltammetric approach for determining the lipophilic vitamin β-carotene was presented by Jashari et al. [41]. The procedure combines a gold disk electrode with square wave voltammetry in acetone as the working medium. It was used for determining the analyte in carrot and sweet potato samples. Acetone was also employed as an extraction solvent in place of the toxic media typically used for lipophilic vitamins. The obtained data were comparable to those of a reference spectrophotometric method.

Cholesterol was detected at a boron-doped diamond electrode using differential pulse voltammetry and an acetonitrile–perchloric acid mixture as the supporting electrolyte [23]. Dairy products were analyzed by combining the newly developed method with liquid–liquid extraction. The results were in close agreement with GC–MS.

A few innovative studies report sensors for in situ analysis (not reported in the Table). Liu et al. [55] proposed a three-electrode-based sensor for simultaneously detecting pyridoxine and melatonin. The sensor was proven effective when inserted directly into an orange, as well as in pureed fruit. The core component of the working electrode is a CuO−poly(L-lysine) coating deposited on a three-dimensional graphene layer. CuO nanoparticles and poly(L-lysine) are able to identify the analytes, while the graphene layer amplifies the catalytic current, increasing the sensitivity. A saturated calomel reference electrode and a Pt counter electrode complete the whole sensor system. Gao et al. [56] described a GC electrode modified with a polydopamine/graphene/MnO_2_ composite for the determination of free tryptophan. The sensor proved reliable for working in tomato fruit and juice. Selectivity, reproducibility, repeatability, and stability were all successfully tested. The platform to be inserted in fruits includes a saturated calomel reference electrode and a Pt counter electrode.

### 2.2. Electrochemical Biosensors for Determining Nutrients

Along with chemically modified electrodes, electrochemical biosensors have also found many applications in the nutritional analysis field. The main difference between biosensors and chemical sensors is the nature of the receptor. In the first case, it is a biomaterial, while in the second case, it is a chemical compound or a group of compounds selectively interacting with the analyte. Electrochemical biosensors offer the combined benefits of electroanalysis and the high specificity of biological substrates. The analytical performance can be further improved using nanomaterials. On the other hand, enzymes and other biological materials can be more costly and difficult to handle than species used in chemically modified electrodes. Table 2 reports a selection of biosensors used for determining food nutrients.

The biosensors proposed for ascorbic acid (vitamin C) determination are both based on GC electrodes modified with unique structures [57,58]. A peptide/metal complex for the direct modification of the GC electrode is reported for the first time by Shao et al. [57]. Coupling the redox properties of the metallocene with the biocompatibility and biomimetic characteristics of the peptides produced a biosensor with superior analytical performance. The presence of the biomaterial effectively catalyzes the oxidation of ascorbic acid, decreasing its anodic overpotential and increasing the peak current compared to the bare GC electrode. Jiang et al. [58] described a novel aptasensor based on polyaniline and gold nanoparticles. Aptamers are known for their superior ability to detect small molecules since they can bind to their targets with high affinity and specificity. In this work, a composite made of polyaniline and gold nanoparticles is first deposited onto the GC surface. Afterward, the aptamer is deposited on the modified electrode and incubated at a controlled temperature to ensure incorporation within the nanocomposite. The resulting platform exhibits a wide linear range and a very low detection limit, and it is able to accurately and selectively determine ascorbic acid. The authors ascribe these properties to the good selectivity and affinity of the aptamer combined with the excellent biocompatibility and electrochemical characteristics of the nanocomposite.

Zhan et al. [59] proposed a GC electrode modified with a heme/nanomaterial composite for the determination of biotin in a wide variety of food samples. The direct electrochemistry of biotin is seldom reported in the literature, due to several limiting factors [59]. The authors suggest an efficient molecular recognition and strong catalytic effect due to the formation of a strongly electroactive supramolecule between heme and biotin, able to overcome the mentioned problems.

Biosensors obtained by combing the relevant enzyme(s) with nanomaterials onto common electrode surfaces were proposed for cholesterol [60], glucose [64]), lysine [69], phosphatidylcholine [71], and sucrose [72].

A cholesterol biosensor based on an enzymatic nanoelectrocatalyst was developed by Arul et al. [60]. The sensing system is actually capable of simultaneously detecting also H_2_O_2_. The enzyme cholesterol oxidase is combined with a bovine serum albumin (BSA)-capped nanocatalyst consisting of gold nanoparticles and a metal-free organic framework. Cholesterol was determined in complex food matrices, such as egg, meat, and dairy products. These samples are challenging due to potential interference from turbidity and many co-existing chemical species. The proposed platform was demonstrated to overcome these difficulties with minimal sample pre-treatment. The results were successfully validated by HPLC-UV.

A simple and sensitive biosensor for detecting glucose in honey was proposed by Xia et al. [64]. In this work, glucose oxidase is loaded on a composite material made of multi-walled carbon nanotubes (MWCNTs) functionalized with β-cyclodextrin. The sensing material is deposited on the surface of a GC electrode, and the detection of the analyte is carried out using amperometry. The proposed platform proved particularly suitable for honey and highly selective as it ruled out the interference of other sugars commonly found in this matrix (galactose, fructose, and sorbose).

Monkrathok et al. [67] proposed an SPE modified with glucose dehydrogenase instead of the more commonly used glucose oxidase. To enhance the electroactive surface area and improve the electron transfer efficiency, graphene oxide and ferrocene-modified linear poly(ethylenimine) were deposited onto the surface. The sensor was integrated into a simple flow injection system for amperometric sensing and applied to the analysis of a sports drink with satisfactory results. Comparison with a standard glucometer demonstrated the method’s reliability.

A unique potentiometric biosensor for detecting lysine in mozzarella cheese was reported very recently [68]. This enzymatic sensor uses the enzyme lysine-α-oxidase (LOx) with a soluble enzyme sensor in combination with an oxygen electrode. Lysine can be selectively determined thanks to its reaction with oxygen and the enzyme. The detailed reaction that takes place at the sensor interface is as follows (Equation (1)):

L-lysine + O_2_ + LOx → α-keto–aminocaproate + H_2_O_2_ + NH_3_ (1)

The electrochemical oxidation reaction occurs at a platinum electrode, and the current output is displayed as oxygen consumed during the L-lactate oxidation reaction. This device provided a very rapid response time (3.5 s) and high storage stability. Moreover, the results were comparable with those obtained by a standard HPLC method.

The phospholipid phosphatidylcholine, commonly known as lecithin, was efficiently determined at a biosensor based on an SPE [71]. The sensor was produced by immobilizing the two enzymes choline oxidase (ChOx) and horseradish peroxidase (HRP) on an SPE with a nanocomposite made of MWCNTs, SnO_2_, and chitosan. The resulting platform was successfully tested in soybean crude oil exhibiting high performance. The method is characterized by speed of analysis, good reproducibility, stability, and accuracy. This last parameter was tested against a reference HPLC method. The same species was also determined at a photoelectrochemical (PEC) biosensor [70]. This sensor was obtained by growing SnO_2_ NPs on an indium tin oxide (ITO) electrode, electropolymerizing polythionine on the surface, and finally immobilizing choline oxidase. The analyte was detected using the developed PEC biosensor under optimal detection conditions, achieving an adequate linear range and low detection limit. The method is characterized by good repeatability, reproducibility, and stability.

A biosensor based on an SPE was proposed for the determination of sucrose [72]. Screen-printed carbon electrodes were modified with a novel enzyme-nanocomposite matrix, created by co-immobilizing invertase (Inv) and glucose oxidase (GOx) in a hydrogel reinforced with Pt nanoparticles anchored to carboxyl-functionalized carbon nanotubes. The analytical features of the sensor were tested on commercial fruit and vegetable juices, and the accuracy was confirmed by a standard method.

**Figure 2:**
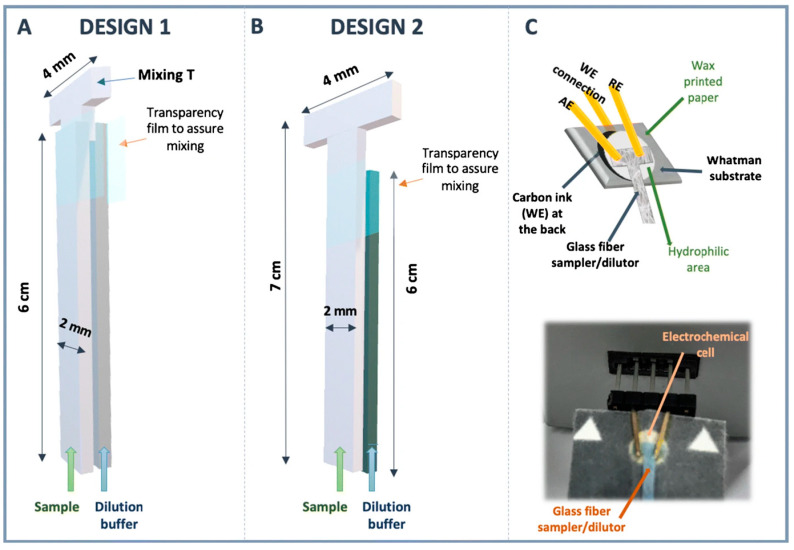
Schematics of the two designs of the lab-on-paper platform for glucose determination: (**A**) design 1, which uses two straight strips and one short “T” piece for mixing, and (**B**) design 2, which uses a long “T”-shaped sampler and a straight strip for dilution. (**C**) Schematic diagram (top) and photograph (bottom) of the assembled microfluidic paper-based device, including gold-plated headers. Reproduced with permission from [62].

Amor-Guiterrez et al. [62] demonstrated the feasibility of a platform capable of rapidly performing different steps of the analytical process using low-cost and sustainable materials. They propose an innovative system that includes a sampling/diluting unit and an electrochemical cell, fully integrated into a single device. A glass-fiber pad takes up the sample and transfers it to a cell consisting of a paper-based carbon-ink working electrode, with gold-plated connector headers acting as reference and counter electrodes. Connection to the potentiostat is made via the pins at the rear part of the connector, which are inserted into the commercial interface. A picture of the proposed device is shown in Figure 2. This lab-on-paper platform was applied to glucose determination in beverages. For this purpose, a biosensor was created by modifying the working electrode surface through the deposition of a mixture of enzymes (GOx and HRP) and a mediator (ferrocyanide). The data obtained for real food samples were compared to those from a spectrophotometric enzymatic kit, demonstrating the accuracy of the proposed method.

**Figure 3:**
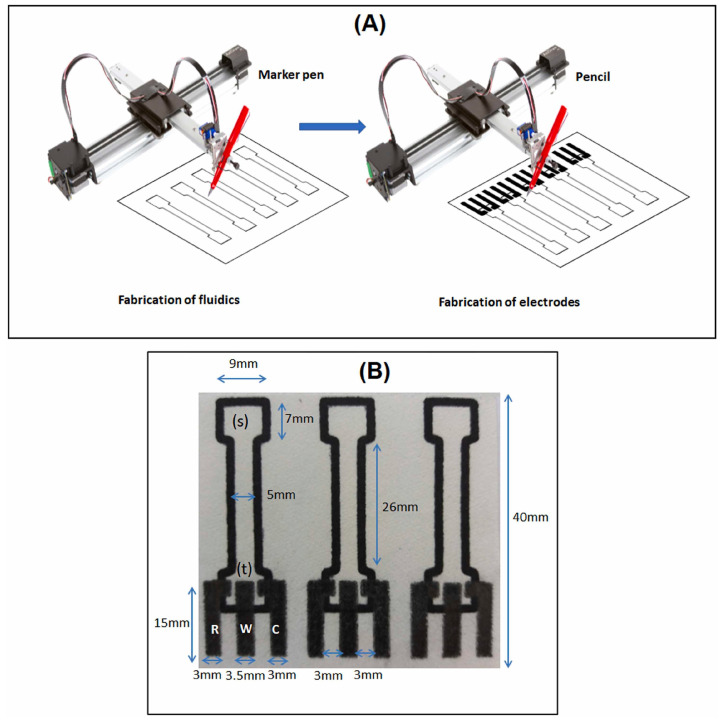
Electrochemical paper-based analytical device (ePAD) for glucose determination. (**A**) Schematic diagram of the fabrication process, (**B**) photographs of three ePADs with dimensions. (s) is the sample zone, (t) is the test zone, W is the working electrode, R is the reference electrode, and C is the counter electrode. Reproduced with permission from [63].

An electrochemical paper-based analytical device (ePAD) was recently proposed as a glucose biosensor [63]. The sensor is fully drawn and fabricated using a high-throughput dual-step approach with ordinary commercial tools. The steps of the fabrication process are reported in Figure 3. The biosensing surface is prepared by depositing a mixture of enzyme (GOx) and mediator (ferrocyanide). Using amperometry, several food samples, such as honey and beverages, were analyzed. The results, when compared to a HPLC method, demonstrated satisfactory accuracy.

A further biosensor for glucose determination in some different food matrices was presented [66]. In this work, GOx was immobilized using plasma-grafted pentafluorophenyl methacrylate as an anchor on a tailored hydrogel covering a titanium dioxide nanotube array as the transducer. Chitosan was also used to protect the enzyme. The platform offers good analytical performance, including repeatability, reproducibility, accuracy, robustness, and long-term stability.

## 3. Phenolic Compounds

Dietary polyphenols, the most abundant antioxidants in the human diet, constitute a heterogeneous class of chemical compounds [73]. They comprise an aromatic ring bearing one or more hydroxyl groups and can be classified using various criteria. Among the most investigated are phenolic acids (caffeic, gallic, etc.) and flavonoids (quercetin, rutin, etc.). Phenolic compounds are secondary plant metabolites present in many fruits and vegetables, as well as in derived foodstuffs (i.e., wine, tea, coffee, and others). Their nutritional value primarily stems from their antioxidant power, which plays a key role in reducing free radicals. However, they also offer many other health benefits.

Polyphenols can be determined individually or as a whole, expressing the result in different ways, such as total phenolic content (TP), antioxidant capacity (AOC), and others. Since some other compounds possess antioxidant properties, the antioxidant capacity/activity usually includes them as well. Cumulative parameters are usually measured using the most abundant compound present in the sample, and the result is expressed as reference species equivalents. Most proposals are based on spectrophotometric techniques but also electrochemistry found a broad consensus. Optical methods are based on the reaction between the antioxidants and free radicals (quenching or scavenging) or on the reaction with transition metals. For this reason, they are influenced by many parameters, such as reaction time, temperature, presence of prooxidants, and other potential interferents. Electrochemical techniques offer low-cost, fast, simple, and sensitive methods in the analysis of phenolic compounds, whether through the scavenging of radicals or by measuring antioxidant capacity itself. In this latter case, electrochemical methods, being based on oxidation or reduction, are simpler and more direct. Moreover, they are not troubled by color interferences, unlike optical methods. This is particularly relevant since most antioxidant-rich foods are strongly colored (dark fruits, red wines, etc.). On the other hand, because of possible synergistic effects among antioxidants, isolating and quantifying individual components (as done by chromatography) could be limiting, especially when a global activity of the sample is required.

### 3.1. Electrochemical Sensors for Determining Phenolic Compounds

A noticeable number of electrochemical sensors have been developed for the determination of individual phenolic compounds and/or global antioxidant-related parameters in foods, as reported in Table 3.

Direct electrochemical oxidation of polyphenols on classical carbon-based electrodes is relatively easy to achieve, and it depends on their chemical structure. The sensitivity of detection can be increased, as in the case of nutrients, by incorporating catalytic materials such as nanostructures. Selectivity can be improved by using molecularly imprinted polymers or by developing enzyme-based biosensors. As in the case of nutrient analysis, the most used electrode substrate is glassy carbon. In a very peculiar case, an unmodified glassy carbon electrode was used for the simultaneous determination of caffeic and gallic acid [82]. The study successfully quantified these phenols in foods by generating second-order data from differential pulse voltammetry conducted at various potential steps and then applying multivariate curve resolution with alternating least squares. Comparison with a standard HPLC method confirms the practicality of this procedure in the wine industry.

Nanomaterials, especially graphene-related ones [74,75,77,90], are the most chosen modifiers, as in the case of nutrients. This is expected since nanostructures are characterized by good electrocatalytic activity toward several species and by the other above-mentioned appealing properties.

Some methods based on GC electrodes modified with graphene species were proposed for caffeic acid determination [74,75,77,78,90]. Manikandan et al. [74] used fluorine-doped GO as a modifier and differential pulse voltammetry as the technique. They demonstrated a strong anti-interference capability in the presence of other hydroxycinnamic acids and ascorbic acid. The modified electrode was used for detecting caffeic acid directly in wine samples without any pretreatment. Another study describes a more complex modifier, i.e., a composite made of flower-like hierarchical graphene/copper oxide and a copper(II) metal–organic framework [75]. The authors demonstrate that this nanocomposite possesses unique structural features, such as high specific surface area and good conductivity, exhibiting excellent electrocatalytic activity for caffeic acid oxidation. Analysis of wine samples and comparison with a reference HPLC method proved the effectiveness of the method. A further caffeic acid sensor was proposed by Magerusan et al. [77]. In this case, the electrode surface was modified using a sulfur-doped graphene material. Interestingly, the synthesis of the nanomaterial was carried out without the use of any organic solvent. Caffeic acid was determined using cyclic voltammetry in coffee samples without any relevant interference by similar phenolic compounds. The same sensor was also applied to the determination of gallic acid in teas with the same analytical performance [90].

A sustainable nanocomposite based on graphene and gold nanoparticles was proposed by dos Santos et al. [78]. A banana pulp extract was used as the precursor for the green synthesis of gold nanoparticles instead of toxic substances that are frequently used. The modified GC electrode was demonstrated to be effective for determining caffeic acid in coffee samples, and the results were comparable to those obtained using UV–Vis spectrometry.

Some methods were based on nanocomposites of carbon nanotubes with other materials [80,97,107]. A GC working electrode was modified with MWCNT and TiO_2_ nanoparticles, using Nafion as a binder [80]. The sensor was proposed for the determination of antioxidants in olive oil. Interestingly, the method uses a green medium for the extraction of polar antioxidants from the samples, i.e., a deep eutectic solvent. In this way, the use of pollutant organic solvents is avoided. Caffeic and vanillic acid were successfully quantified by means of square wave voltammetry, and the results were compared with those of a reference spectrophotometric method. A novel nanocomposite made of Au–Co bimetal nanoparticles embedded in nitrogen-doped CNT hollow polyhedron was used as a modifier for the determination of quercetin [97]. The authors demonstrated that the composite exhibited synergistic effects from the components, including large specific surface area and high electrochemical activity toward the analyte. Quercetin was successfully determined in onion samples. However, it was pointed out that similar flavonoids might interfere due to the close peak potentials. MWCNTs were also functionalized with Cu(II)-neocuproine and carrageenan and utilized to modify a GCE for evaluating the antioxidant capacity [107]. In this case, AOC was expressed as Trolox equivalents, and the food samples were herbal teas, plant extracts, and fruit juices. Comparison with a standard CUPRAC test confirmed the method’s reliability.

A GC electrode modified with a magnetite nanoparticles–zeolitic imidazolate framework material was used for the determination of coumaric acid in fruit juices [92]. The nanocomposite possesses a high surface area and a well-designed shape, which enhance the sensor’s sensitivity and selectivity. The sensor also shows satisfactory repeatability and stability. The same author proposed also a sensor for rutin, based on a newly designed layered MAX phase as the modifier [102]. MAX phase structures are transition metal carbides/nitrides widely used in developing sensors due to their valuable properties [102]. The MAX phase structure (Ti_3_Al_0.5_Cu_0.5_ C_2_) was synthesized starting from the metals and C powders, then suspended in dimethylformamide, and finally drop-casted onto the electrode surface. The obtained sensor exhibited a wide linear range, high selectivity, good reproducibility, acceptable stability, and satisfactory sensitivity. It was tested in fruit samples obtaining results in agreement with an HPLC method.

A different nanocomposite with magnetite particles was prepared by Saljooqi et al. [93]. Fe_3_O_4_ nanoparticles were coated with inert silica to prevent aggregation and combined with a conductive polymer (polyaniline) and gold nanoparticles. The resulting composite nanomaterial was drop-casted onto a GC electrode. The synergic effect of the components made the obtained sensor a good option for determining quercetin not only in food samples (tea and apple juice) but also in biological matrices.

A novel nanocomposite made of a nanostructured conductive polymer, an ionic liquid (IL), and palygorskite, was recently synthesized for developing a gallic acid sensor [87]. This nanocomposite combines the electrocatalytic ability of the polymer, the increased electron transfer rate of the IL, and the high adsorption capability of palygorskite (a magnesium–aluminum silicate mineral). The resulting sensor presented good stability, repeatability/reproducibility, and selectivity. The practical usefulness was demonstrated by accurately determining gallic acid in tea and fruit juices.

Porous carbon-based materials have also attracted increasing interest [76,89]. A GC electrode modified with a nanocomposite of Super-P carbon black (SPCB) and mesoporous silica was applied to the analysis of gallic acid [89]. The newly synthesized material combines the appealing properties of its components: SPCB possesses excellent electrical conductivity and significantly improves the charge transfer efficiency, while the mesoporous structures improve the surface accumulation of the analyte. The sensor presented good repeatability, reproducibility, and anti-interference properties. Analysis of tea samples demonstrated the effective applicability of the method.

Environmentally friendly porous materials were also proposed [76]. Porous carbon products were obtained by direct carbonization of *Tetrapanax papyriferus* at different temperatures (600, 700, 800, and 900 °C). GC electrodes were modified with suspensions (in dimethylformamide) of the obtained materials. According to the authors, the high porosity facilitated the adsorption of the analytes and ion transport. Voltammetric analysis of caffeic acid in coffee and wine samples demonstrated the satisfactory analytical features of the proposed method.

Molecular imprinting technology has been extensively used in the development of sensors for polyphenols [85,86,88,99,101]. The Molecular Imprinted Polymer (MIP) technology involves the synthesis of polymers with specific binding sites complementary to the target analyte (template). After the removal of the template, the material retains binding sites that match the analyte. Consequently, MIPs impart high specificity/good selectivity to the sensor, and this feature is particularly useful when dealing with mixtures of species with analogous chemical structures, such as phenolic compounds. When used as an electrode modifier, they are a valid alternative to biological species since they are generally cheaper, more stable, and easier to handle. Gao et al. [85] proposed a MIP-modified GC electrode for determining curcumin. The authors designed a novel functional monomer (a chitosan oligosaccharide), and they obtained a MIP membrane through in situ bulk polymerization followed by electrochemical polymerization on the electrode surface. It was demonstrated that the obtained membrane had superior affinity for the template molecule, increasing the specificity and detection sensitivity of the designed sensor compared to previously reported MIPs. The relevant method was successfully applied to turmeric extracts.

MIP technology was also used for manufacturing a potentiometric sensor for gallic acid [88]. The imprinted polymer was obtained using a bulk polymerization approach. The MIP particles were mixed with a plasticizer and other compounds to obtain the final disk membrane to be used as a sensing element in the potentiometric sensor. The proposed device was applied to gallic acid determination in dried extracts of some fruits, using HPLC as the reference method. The simplicity, portability, low cost, sensitivity, and low energy input make this method a valid alternative to more popular ones.

Recently, hybrid MIPs with magnetic properties were also proven as valid substitutes for biologically modified magnetic particles due to their lower cost and higher robustness. An example is the curcumin sensor described by Li et al. [86]. In this work, magnetic Fe_3_O_4_ nanoparticles were combined with the monomer zein and the template molecule to obtain the imprinted polymer via self-polymerization at room temperature. Comparing the electrochemical behavior of curcumin at the MIP-modified electrode with those of a bare one and a MIP(without nanoparticles)-modified one, the authors demonstrated enhanced sensitivity and selectivity due to the synergistic effect of the components. The sensor was successfully used for determining curcumin in potato chips.

MIP sensors for resveratrol were also designed [99,101]. In both cases, the synergic action of a MIP and nanoparticles was exploited. Huang et al. [99] used polyacrylamide (PAM) as the recognition polymer and polyaniline (PANI)/gold nanoparticles to enhance the sensor’s sensitivity. The fabrication of the MIP-modified electrode was a multi-step procedure: the first step was the electrodeposition of the nanoparticles onto the electrode, followed by the electropolymerization of PANI, then of PAM in the presence of the template, a crosslinking agent, and a catalyzer. Finally, the template molecules were completely removed. A similar procedure was used for developing another MIP-based sensor for resveratrol [101]. In this case, the chosen recognition polymer was PAM, while a nanocomposite of graphene–gold nanoparticles was used to enhance sensitivity. In both cases, the obtained sensors were tested in red wine samples with satisfactory results, comparable to those obtained by standard HPLC methods. Despite the similarities in the sensor development, linear ranges and LODs differed significantly.

Quercetin was detected by using a poly (safranine O)-modified GC electrode [94]. The modifier layer was obtained by direct electropolymerization of the monomer onto the electrode surface. Its high electrocatalytic activity toward the analyte was demonstrated by cyclic and square wave voltammetry experiments. The proposed sensor was used for determining quercetin in wine and fruit juice with satisfactory accuracy. Another polymer-modified electrode was designed for the same compound [95]. In this case, a poly(chromotrope fb)-modified activated pencil graphite electrode (PGE) was described. PGEs have some interesting advantages such as low cost, mechanical robustness, renewability, and ease of modification. After activation by cyclic voltammetry, the PGE was modified by electropolymerization in a monomer solution using cyclic voltammetry. Various food samples, including vegetables and fresh and dried fruits, were analyzed using the prepared electrochemical platform and a standard spectrophotometric method. The results demonstrate the good accuracy and precision of the newly proposed method.

Several methods based on SPEs have also been applied to the quantification of phenolic compounds in foods [83,84,91,98,100,109].

Carbon black/molybdenum disulfide nanohybrid screen-printed electrodes were proposed for the determination of cocoa catechins [83] and of two olive oil *o*-diphenols (hydroxytyrosol and oleuropein) [91]. The combination of the nanomaterials exhibits low charge-transfer resistance and high electrical conductivity, improving electrocatalysis and the charge-transfer ability compared to the individual materials. SPE modification was performed by drop-casting the mixed nanomaterials dispersion. Results obtained from analyzing real samples were compared with reference methods, demonstrating the efficacy of the proposed platform in very complex food matrices.

Another porous nanostructured material, ordered mesoporous carbon (OMC), was used as a modifier for an SPE-based sensor [100]. The optimized sensor was used for determining resveratrol in red wine samples. Due to its high surface area and conductivity, OMC enhanced the electron transfer between the analyte and the electrode surface. Therefore, its analytical performance was much better than that of the bare SPE. The OMC/SPE also showed excellent anti-interference ability in a matrix that could be difficult to handle. In fact, the dark color of red wine is usually very challenging for spectrophometric techniques.

Chlorogenic acid (CGA) [84] and quercetin glucosides [98] were determined by using carbon nanotube-modified SPEs. In the first case [84], a novel potentiometric sensor was fabricated by modifying an SPE platform with a sensing membrane. The membrane comprises a [Ni^II^(bathophenanthroline)_3_][CGA]_2_ complex as the sensory material and MWCNTs as the sensitivity enhancer. The analytical features of the potentiometric method were carefully assessed with satisfactory results. The sensor was applied for the analysis of CGA in some green coffee extracts and fruit juices. The results were in agreement with those obtained by a standard HPLC method.

For the analysis of quercetin glucosides, a long-length CNT-based thin-film SPE was developed [98]. The working electrode surface was modified by dropping CNTs dispersed in a carboxymethylcellulose (CMC) aqueous solution. The use of CMC favored a network of long-length CNTs that was much denser than in the case of common CNTs. The resulting sensor showed a well-defined and reproducible signal, low background current, high stability, and sensitivity toward quercetin glucosides. Determination of these individual compounds was carried out in a variety of food matrices. The results were in good agreement with those obtained by HPLC.

Martin et al. [109] boosted the performance of an SPE with iron tetrasulfonated phthalocyanine (FeTsPc). The sensor was intended for the determination of the total phenolic content in mate tea. Preliminary experiments were conducted to select the optimal pH of the FeTsPc solution. All the tested solutions showed a decrease in the oxidation potential of the model species (catechol) compared to the unmodified electrode. The optimized sensor results were compared with the standard Folin–Ciocalteu assay. The minimal differences were explained as related to the chemical structure of polyphenols in mate herb: the standard assay determines the total polyphenol content, while the electrochemical methods determine only the polyphenols that are oxidized at the potential fixed by the method itself.

Two photoelectrochemical (PEC) sensing platforms were proposed for the determination of quercetin [96] and rutin [103]. Multi-walled carbon nanotubes, a polymer composite, and semiconducting BiVO_4_ were used to prepare a photosensitive ITO-based sensor for quercetin detection [96]. The ITO base was modified by simply drop-casting a mix of the dispersed modifiers. The developed sensor demonstrated high sensitivity, good reproducibility, long-term stability, and high selectivity toward quercetin sensing. It was then used for black tea samples, obtaining results comparable to a classic HPLC method. Bakhnooh and Arvand [103] developed a novel signal-off type PEC sensor based on TiO_2_ nanotube arrays (TNT) decorated with mixed metal sulfide semiconductor nanoparticles for rutin detection. The nanoparticles were directly deposited on the surface of TNT via a one-step electrodeposition approach followed by an annealing process. The appealing synergistic effect of the components was demonstrated by several experiments. For detection purposes, the photocurrent response of the fabricated sensor was recorded by amperometry. The constructed PEC sensor was successfully applied to detect rutin in fruits.

Another modified ITO electrode was designed for the voltammetric determination of several antioxidants (caffeic and gallic acid, but also ascorbic acid) [81]. In this work, hybrid copper–phthalocyanine/conjugated graphitic carbon nitride nanosheets were synthesized by a sonochemical method. The ITO electrode covered with this composite nanomaterial showed excellent electrocatalytic activity toward the target antioxidants. Some beverages, such as fruit juices, wines, and teas, were analyzed with minimal pretreatment, and the results were confirmed by a standard HPLC method.

Gevaerd et al. [105] described a carbon fiber ultramicroelectrode for the determination of the antioxidant capacity (AOC) of wine and grape, expressed as caffeic acid equivalents. The electrode was constructed using an arrangement of carbon fibers assembled in a glass capillary. Real sample analysis results were compared with the Folin–Ciocalteu assay as the reference method. The higher values obtained by the reference method were explained as being due to the presence of interfering species, such as sulfur dioxide and sugars that react with the Folin–Ciocalteu reagents. Despite these differences, the same trend in antioxidant activity was observed in the wine samples. Some experiments involving the direct insertion of the sensor into grapes paved the way for in situ analysis.

Antioxidant capacity was also tested by quantifying the consumption of the 2,2-diphenyl-2-picrylhydrazyl (DPPH) radical at laser-induced graphene (LIG) electrodes [108]. The method is based on amperometric detection in a batch injection system. The results obtained in tea samples were compared with the conventional spectrophotometric DPPH test, confirming the sensing abilities of the LIG electrodes.

**Figure 4:**
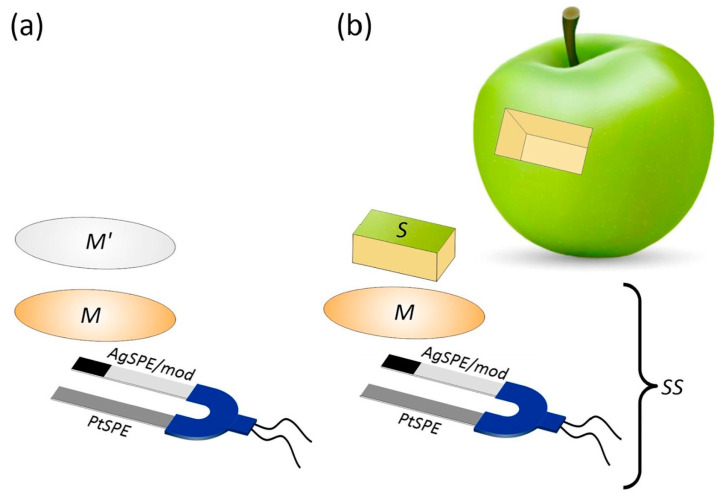
Sensing platform for AOC assessment: (**a**) sensor system for model antioxidant determination; (**b**) sensor system for samples. M: membrane impregnated with a solution of the mediator; M′: membrane impregnated with the model antioxidant solution; S: slice of the test sample; PtSPE: platinum screen-printed electrode; AgSPE/mod: modified silver screen-printed electrode; SS: sensor system. Reproduced with permission from [110].

An additional sensing platform for in situ analysis was developed [110] (not reported in the Tables). It is a contact hybrid potentiometric AOC sensor consisting of two SPEs and a membrane impregnated with a mediator (K_3_[Fe(CN)_6_]/K_4_[Fe(CN)_6_] in phosphate buffer). The working electrode is a platinum SPE, while the reference electrode is a silver SPE modified by a mixed precipitate of AgCl and ferricyanide. The whole system is shown in Figure 4. Measurements were performed by connecting the sensor to a portable potentiometric analyzer. The proposed method was tested in a wide variety of fruit and vegetable samples. The results showed high reproducibility and good accuracy.

### 3.2. Biosensors for Determining Phenolic Compounds

Unlike nutrients, biosensors for phenolic compounds are less explored due to their high inherent electroactivity, which allows for efficient detection even at enzyme-free sensors, as reported in the previous paragraph. Nevertheless, some promising biosensors (Table 4) have been proposed for analyzing individual compounds [111,112] or related cumulative parameters [113,114,115] even in complex food matrices like edible oil.

A novel composite material was used for modifying a GC electrode along with laccase [111]. The resulting biosensor was used for determining some polyphenols. The proposed material is a conductive composite made of polymers and nanoparticles. The enzyme is immobilized on its surface, using glutaraldehyde as a linker. Voltammetric tests showed that the developed biosensor exhibits catalytic activity toward the oxidation of polyphenols. Laccase was regenerated through direct electron transfer between the electrode and the enzyme. Analytical parameters were assessed for the determination of catechol, gallic acid, and caffeic acid, but only gallic acid was found in a real sample (white wine).

Apetrei et al. [112] proposed the use of a crude extract instead of the pure enzyme. This solution is particularly interesting, as it eliminates the need for costly and laborious processes of enzyme separation and purification. A crude polyphenol oxidase (PPO) extract is self-encapsulated within polypyrrole (Ppy) biosynthesized in situ. The amperometric response of the obtained biosensor to some common phenols (catechol, gallic acid, caffeic acid) was analyzed, and catechol was chosen as the most suitable analyte to determine in real food samples. The performance of the biosensor was compared to that of similar ones to study the influence of the newly proposed modifier. The results confirm that the bio-Ppy biosensor exhibits very similar characteristics to a chemically synthesized one, rendering the biocatalytic synthesis a promising approach. Real samples of fruit wines were analyzed with the proposed biosensor and the standard Folin–Ciocalteua assay, showing a good agreement.

Zrinski et al. [113] described the evaluation of the antioxidant capacity of beverages using a laccase biosensor. The enzyme is immobilized onto a nanocomposite-modified SPE. In this case, the used nanostructures are graphene nanoplatelets and gold nanoparticles. The biosensor showed good repeatability, reproducibility, long-lasting stability, and excellent electrocatalytic activity toward oxidation of hydroquinone. It was used for the analysis of wine and fruit syrup. The results are consistent with those of the standard Trolox Equivalent Antioxidant Capacity (TEAC) assay.

Two biosensors were developed for determining the total phenolic content of olive oil [114] and beers [115]. In the first case [114], the authors propose a laccase-based biosensor obtained by encapsulation of the enzyme in a chitosan/galactomannan composite. The advantages of this encapsulation are the preservation of the enzyme and the reduction of interferences, maintaining a good charge transfer rate. Analysis of olive oil samples shows that this method is a valuable alternative to the standard Folin–Ciocalteu assay. In the other paper [115], the authors explored the applicability of miniaturized and portable biosensing technology to the estimation of the total phenolic content of beers. They developed a tyrosinase amperometric biosensor immobilizing the enzyme by crosslinking on a gold nanoparticle-modified SPE. Also, in this case, the results were validated by comparison with the standard Folin–Ciocalteu assay.

## 4. Conclusions and Future Perspectives

**Figure 5:**
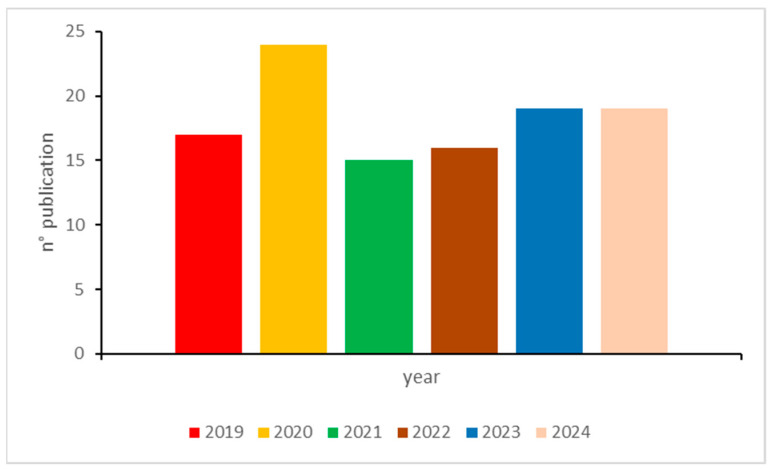
Graph reporting the number of reviewed publications divided by year.

This paper reviewed recent innovations in the electroanalysis of food nutritional and bioactive components made by sensors and biosensors. As shown in Figure 5, the number of applications has remained steady and significant in recent years.

Several new materials were proposed as modifiers to increase the selectivity, sensitivity, and conductivity of the electrode substrate. Among those, the large majority is represented by nanocomposites, especially based on carbon nanostructures. Many novel nanomaterials have shown good electrocatalytic abilities. It is worth mentioning variously shaped nanoparticles of metals, transition metals, and their oxides. Porous materials were also used in combination with nanostructures to increase analyte adsorption.

There has been growing attention to environmentally friendly materials and greener methodologies for the development of the sensor and the sample treatment. Examples of this inspiring trend include paper-based sensors, alternative media to organic solvents, and materials derived from natural sources.

Multianalyte determination was performed through two approaches: simultaneous quantification of the species or as a cumulative parameter. Individual quantification has been less explored and remains challenging. However, some studies suggest that advanced chemometric tools can be beneficial in addressing the difficulties posed by overlapping electrochemical signals.

The numerous contributions demonstrate that electroanalysis could represent an attractive alternative to more common techniques, mainly due to the faster response and lower cost. Many proposals have addressed the challenges of the food industry by providing high analytical accuracy even in difficult matrices. Foodstuffs of various complexities were investigated: beverages, water-rich produce, dairy products, dry samples such as flours and cereals, edible oils, and meats. Many described methods required little to no sample pretreatment. In this regard, particularly remarkable is the development of in situ sensors.

It is also important to remember that electrochemical instruments, differently from other devices, do not require additional components to detect the signal and that the detection itself can be directly integrated into the circuit. Coupled with the development of portable instruments and disposable parts, this paves the way for decentralized testing in routine analysis. Nowadays, there is a growing demand for cost-effective, portable screening devices outside the laboratory. Consumers’ increasing interest in conscious food choices is driving the need for practical, real-time analytical devices in everyday life.

It should also be pointed out that this review only covers methods already tested on real samples and whose accuracy has been verified by comparison with a reference method. However, dozens of additional promising studies are reported in the literature. Therefore, many further procedures could be implemented in the coming years.

## Figures and Tables

**Table 1:** Main characteristics of the sensors used for determining food nutrients.

Analyte	Sample	Sensor (Technique)	Linear Range/LOD	Ref.
**AMINO ACIDS**				
Phenylalanine	Lemonade, sugar	MnO_2_/N-RGO/GCE (DPV)	15–220/3.59 µM	[5]
Tryptophan	Milk, chocolate	ND/Au NP/PEDOT:PSS/SPE (SWV)	0.8–18/0.2 µM	[6]
Tryptophan	Milk, flour	Ag-zeolite/CPE (DPV)	0.02–1.2 µM/6.3 nM	[7]
Tryptophan	Sunflower and pumpkin seeds	HAp-GO/SPE (LSV)	7–1000/5.5 µM	[8]
Tryptophan	Egg white, milk	(BTPB) SiW_11_O_39_Ni/P-ERGO/GCE (CA)	1–2000/0.83 µM	[9]
Tryptophan	Egg white	BCN-SPE (DPV)	1–400/0.036 µM	[10]
L-tyrosine	Meat floss	PEI-P_2_Mo_16_V_2_-IL-CNT/ITO (CA)	0.58 µM–0.12 mM/0.17 µM	[11]
L-tyrosine	Egg	ZnO NP-MIP/GCE (DPV)	4–1100/0.008 µM	[12]
**CARBOHYDRATES**				
Fructose	Honey	Ni(II)-2,3dhS-CNT/SPE (CV)	48–130/16 µM	[13]
Glucose			36–130/12 µM	
Glucose	Beverages	PtNi@RGO/GRE (CA)	0.02–5 mM/6.3 µM	[14]
Glucose	Drinks	NiCo_2_O_4_ NR-NP/Ni foam (CA)	0.4–5200/1.1 µM	[15]
Glucose	Beverage	Mn–NiO NS/C cloth E (CA)	2 µM–2.14 mM/0.23 µM	[16]
Glucose	Beverages	Cu_2_O micro-nano cubes/Nafion/GCE (CA)	0.05–10.65 mM/0.87 µM	[17]
Glucose	Beverage	NiCo-LDH NS/Ni foam (CA)	0.4–50 µM/48.76 nM	[18]
Glucose	Beverages	WO_3_@TNT (CA)	1–6.5 mM/0.19 mM	[19]
Glucose	Apple juice	cMWCNT/AuNP/SPE (CA)	5–80/0.537 mM	[20]
Fructose			2–20/1.630 mM	
Arabinose			2–50/1.811 mM	
Mannose			5–60/4.903 mM	
Xylose			2–40/0.693 mM	
Galactose			5–40/2.105 mM	
Total sugars (as glucose)	Fruit juice	NiO NP/GCE (CA)	1.25–600/0.38 µM	[21]
**FATS**				
Cholesterol	Corned beef	citrate- β-cyclodextrin-MNP + SPE (CA)	0–100/3.93 μM	[22]
Cholesterol	Dairy products	BDDE (DPV)	10–100/4.9 μM	[23]
**MINERALS**				
Ca^2+^	Milk powder	CPE1 (POT)	1–1000/3.33 µM	[24]
		CPE2 (POT)	0.1–1000/0.3333 µM	
K^+^	Drinks, orange juice	ISM-RGO/AuE (POT)	0.06–250/0.06 mM	[25]
K^+^	Milk powder, juice	ISE (POT)	0.01–100 mM/5.88 µM	[26]
K^+^	Apple juice, milk, soybean, coconut water	SPE (POT)	10 µM–0.1 M/10 µM	[27]
Fe^3+^			1 µM–0.1 M/1 µM	
Iron	Spinach	Iodine/Pt-RDE (HV)	0.4–100/0.07 ppm	[28]
Iron	Milk powder	CNP/MBG (SWV)	0.3–5.9/0.18 mg L^−1^	[29]
Phosphate (as P)	Beef, beans, garlic, dried apricots	ISE (POT)	1 µM–0.1 M/0.24 µM	[30]
Selenium	Tea, sprouts, peanuts	Au NC-FGR/GCE (SWASV)	0.002–5 mg L^−1^/0.27 µg L^−1^	[31]
Selenium	Rice, egg	AuHD/GCE (SWASV)	50–700/1.4 nM	[32]
**VITAMINS**				
Ascorbic acid	Fruit juice	P_2_Mo_17_V/Ru(bpy)_3_/CS-Pd/ITO (CA)	0.125–118/0.1 µM	[33]
Ascorbic acid	Fruit juice	PAP/ZrO_2_ NP/CNT/GCE (SWV)	1 –295/0.35 µM	[34]
Ascorbic acid	Juice	SPEs (CV)	50–2000 µM/0.0022–0.021 mg 100 mL^−1^	[35]
Ascorbic acid	Milk beverages	RGO/GCE (CA/FIA)	10–50/4.7 µM	[36]
Ascorbic acid	Juice powder, soy and baby milk	MWCNT-hybrid/GCE (CA)	5–4000/1.5 nM	[37]
Ascorbic acid	Beverages	AuNP/PGSE (SWV)	5–100/1.86 µg L^−1^	[38]
Ascorbic acid	Beverages	CNT-Co/CF@NC (SWV)	20–1400/1 µM	[39]
Astaxanthin	Crustaceans	paraffin-GRE (SWV)	50–400/15.77 µmol dm^−3^	[40]
β-carotene	Vegetables	AuE (SWV)	6–590/1.6 µM	[41]
Folic acid	Fruits, vegetable, flour	Magnetic-MIP/CPE (SWV)	2–12/0.1 µM	[42]
Folic acid	Fruit juice, coconut water	3D Printed C black E (SWV)	10–200/5.1 µM	[43]
Folic acid	Fruit juice	Co@CNT/GCE (DPV)	0.0050–10 µM/2.3 nM	[44]
Folic acid	Fruits, vegetables, rice	MIP/GE (DPV)	20–400/0.018 µM	[45]
Folic acid	Breakfast cereals, cocoa drinks	Ni-zeolite/C black/GCE (DPV)	0.004–0.22 mg L^−1^/1.3 µg L^−1^	[46]
Niacin			0.15–10/0.045 mg L^−1^	
Riboflavin			0.008–0.24 mg L^−1^/2.3 µg L^−1^	
Vit. B_12_			0.003–0.1 mg L^−1^/0.92 µg L^−1^	
Riboflavin	Milk powder, malt drink	Poly (glutamic acid)—ZnO NP/CPE (SWV)	0.005–10/0.0007 µM	[47]
Vit. A, D, E, K	Infant milk, yogurt, parsley	GR-Nafion/SPE (SWAdSV)	0.02–1/0.0086, 0.0063, 0.0075, 0.0071 µg mL^−1^	[48]
Vit. A	Milk	β-cyclodextrin/MWCNT/GCE (SWV)	8–100/2.5 µM	[49]
Vit. D_3_			0.8–60/0.67 µM	
Vit. E			0.5–60/0.48 µM	
Vit. K_1_			0.1–20/0.09 µM	
Vit. B_2_	Beverages	N,S-GQD-CS/GCE (SWASV)	0.001–8.0 μM/0.30 nM	[50]
Vit. B_6_			0.1–18.0 μM/30.1 nM	
Vit. B_12_			0.001–8.0 μM/0.32 nM	
Total Vit. K	Green vegetables	SPGE (SWASV)	1–15/0.099 µg mL^−1^	[51]

Modifiers: AuHD = hierarchical dendritic gold nanostructure; BTPB = 1,4-bis(triphenylphosphonium)butane; cMWCNT = carboxylated multi-walled carbon nanotubes; CNP = carbon nanoparticles; CNT = carbon nanotubes; CS = chitosan; ERGO = electrochemically reduced graphene oxide; FGR = fluorinated graphene; GO = graphene oxide; GQD = graphene quantum dots; GR = graphene; HAp = hydroxyapatite; IL = ionic liquid; ISM = ion selective membrane; LDH = layered double hydroxide; MIP = molecularly imprinted polymer; MNP = magnetic nanoparticles; MWCNT = multi-walled carbon nanotubes; NC = nanocages; ND = nanodiamonds; NP = nanoparticles; NR = nanorods; NS = nanosheets; PAP = poly(aminopyrazine); PEDOT:PSS = poly(3,4-ethylenedioxythiophene):polystyrene sulfonate; PEI = polyethylenimine; RGO = reduced graphene oxide; TNT = titanium dioxide nanotube arrays. Electrodes: BDDE = Boron-doped diamond; CPE = carbon paste; GCE = glassy carbon; GE = graphite; GRE = graphite rod; ISE = ion selective; ITO = indium tin oxide; MBG = modified borate glassy matrix; PGSE = pyrolytic graphite sheet; RDE = rotating disk; SPE = screen-printed; SPGE = screen-printed graphene. Techniques: CA = chronoamperometry; CV = cyclic voltammetry; DPV = differential pulse voltammetry; FIA = flow injection analysis; HV = hydrodynamic voltammetry; LSV = linear sweep voltammetry; POT = potentiometry; SWAdSV = square wave adsorptive stripping voltammetry; SWASV = square wave anodic stripping voltammetry; SWV = square wave voltammetry.

**Table 2:** Main characteristics of the biosensors used for determining food nutrients.

Analyte	Sample	Sensor (Technique)	Linear Range/LOD	Ref.
Ascorbic acid	Beverages	FPC-Cu(II)/GCE (DPV)	0.1–25/0.02 µM	[57]
Ascorbic acid	Fruit, vegetables, milk powder	MCH/Apt/Au NP/PANI/GCE (DPV)	1–10^5^/0.10 ngL^−1^	[58]
Biotin	Milk, flour, juices, egg	heme/Au NP/GCE (DPV)	5–50,000/1.6 nM	[59]
Cholesterol	Meat, egg yolk, dairy products	ChOx-Au NP-MFOF/GCE (CA)	250 nM–3 mM/53.2 nM	[60]
Glucose	Orange juice, cola beverage	ePAD (GOx-HRP) (CA)	0.5–300/0.4 mM	[61,62]
Glucose	Honey, beverages	ePAD (GOx) (CA)	0.10–100/0.08 mM (LOQ)	[63]
Glucose	Honey	GOx/β-cyclodextrin/MWCNT/GCE (CA)	50–1150/0.42 µM	[64]
Glucose	Beverages, sauces, dairy products	CS-GOx/pgPFM/HEMA-co-EGDA/TNT/Ti (CA)	0.25–1.49/0.10 mM	[65,66]
Glucose	Sport drink	GDH/PEI-Fc/GO/SPE (CA/FI)	1–40/0.28 mM	[67]
Lysine	Cheese	LOx/O_2_ E (POT)	30–1300 µM/0.03 mM	[68]
Lysine	Milk	LOx NP/AuE (CA)	10–800/10 µM	[69]
Phosphatidylcholine	Soybean oil	ChlOx/PTh/SnO_2_ NP/ITO (PEC)	0.03 –4/0.005 mM	[70]
Phosphatidylcholine	Soybean crude oil	ChlOx-HRP/MWCNT-SnO_2_-CS/SPE (SWV)	30–270/3 mgL^−1^	[71]
Sucrose	Fruit and vegetable juice	INV-GOx-MWCNT-Pt NP/SPE (CA)	1 nM–0.1 mM/1 nM	[72]

Modifiers: Apt = aptamer; ChlOx = choline oxidase; ChOx = cholesterol oxidase; CS = chitosan; EGDA = ethylene glycol diacrylate; Fc = ferrocene; FPC = ferrocene–peptide conjugate; GDH = glucose dehydrogenase; GO = graphene oxide; GOx = glucose oxidase; HEMA = hydroxyethyl methacrylate; HRP = horseradish peroxidase; INV = invertase; LOx = lysine oxidase; MCH = 6-mercapto-1-hexanol; MFOF = metal-free organic framework; MWCNT = multi-walled carbon nanotubes; NP = nanoparticles; PANI = polyaniline; PEI = polyethylenimine; pg-PFM = plasma-grafted pentafluorophenyl methacrylate; PPO = polyphenol oxidase; PTh = polythionine; TNT = highly ordered titanium dioxide nanotube arrays. Electrodes: ePAD = electrochemical paper-based analytical device; GCE = glassy carbon; ITO = indium tin oxide; SPE = screen-printed. Techniques: CA = chronoamperometry; DPV = differential pulse voltammetry; FI = flow injection; PEC = photoelectrochemical detection; POT = potentiometry; SWV = square wave voltammetry. LOQ = limit of quantification.

**Table 3:** Main characteristics of the sensors used for determining phenolic compounds.

Analyte	Sample	Sensor (Technique)	Linear Range/LOD	Ref.
Caffeic acid	Wine	F-GO/GCE (DPV)	0.5–100/0.018 µM	[74]
Caffeic acid	Wine	MOF/GCE (DPV)	0.02–10 µM/7 nM	[75]
Caffeic acid	Wine, coffee	Porous C/GCE (DPV)	0.01–10 µM/5.34 nM	[76]
Caffeic acid	Coffee	S-doped GR/GCE (CV)	0.1–100/0.0303 µM	[77]
Caffeic acid	Coffee	GR-Au NP-bex/GCE (SWV)	0.05–10 µM/16 nM	[78]
Caffeic acid	Wine, fruit juice	ZIF NP/Au@Co_3_O_4_@CeO_2_/GCE (DPV)	0.001–3/0.00012 µM	[79]
Caffeic acid	Olive oil	Nafion/TiO_2_/MWCNT/GCE (SWV)	0.028–1.1 mM/1.82 µM	[80]
Vanillic acid			0.03–1.19 mM/5.32 µM	
Caffeic acid	Fruit juices, tea, wine	CuPTc/g-C_3_N_4_NS/ITO (DPV)	0.05–240/0.008 nM	[81]
Gallic acid			10–185/0.5 nM	
Caffeic acid	Red wine	GCE (DPV)	7.6–300/7.6 mg L^−1^	[82]
Gallic acid			1.6–300/1.6 mg L^−1^	
Catechins	Cocoa powder	C black-MoS_2_/SPE (DPV)	0.12–25/0.17 µM	[83]
Chlorogenic acid	Green coffee, fruit juice	MWCNT/SPE (POT)	0.1–1000/0.07 µM	[84]
Curcumin	Turmeric	MIP/GCE (DPV)	10 nM–2 µM/5 nM	[85]
Curcumin	Potato chips	MIP-NP/GCE (CV)	0.1–100 µM/10 nM	[86]
Gallic acid	Tea, fruit juice	PmPD/Pal-IL/GCE (DPV)	1–300/0.28 µM	[87]
Gallic acid	Tea, fruits	MIP (POT)	10–320/10 µM (LOQ)	[88]
Gallic acid	Teas	C Black NP@MSi NP/GCE (DPV)	0.5–10 µM/4.911 nM	[89]
Gallic acid	Teas	S-doped GR/GCE (CV)	0.1–100/0.0303 µM	[90]
Hydroxytyrosol	Olive oil	C black-MoS_2_/SPE (DPV)	2–100/1 µM	[91]
Oleuropein			0.3–30/0.1 µM	
p-coumaric acid	Orange juice	Fe_3_O_4_@ZIF/GCE (DPV)	0.5–12/0.18 µM	[92]
Quercetin	Tea, apple juice	MNP-PANI-Au NP/GCE (DPV)	0.01–15 µM/3.8 nM	[93]
Quercetin	Wine, fruit juice	poly(safranine O)/GCE (SWV)	0.01–8/0.005 µM	[94]
Quercetin	Vegetables, fruits	poly(chromotrope fb)/PGE (DPV)	0.01–1.2 μM/1.9 nM	[95]
Quercetin	Tea	MWCNT@PST-PAN@BiVO_4_/ITO (PEC)	10–200/0.133 μM	[96]
Quercetin	Onion	Au-Co-N CNT hollow Polyhedron/GCE (DPV)	0.050–35/0.023 µM	[97]
Quercetin glucosides	Onion, apple, buckwheat	MWCNT/CMC/SPE (CV)	0.018–5/0.0018–0.0030 mg dL^−1^	[98]
Resveratrol	Wine	MIP(PAM-PANI-Au NP)/GCE (DPV)	1–200 µM/87 nM	[99]
Resveratrol	Wine	OMC/SPE (CA)	5–50/0.473 µM	[100]
Resveratrol	Wine	MIP(GR-Au)/GCE (CV)	0.01–10/0.0044 µM	[101]
Rutin	Fruits	MAX phase/GCE (DPV)	0.02–50/0.015 µM	[102]
Rutin	Fruits	Cu_2_SnS_3_/TNT/Ti (PEC)	0.001 nM–100 µM/0.0007 nM	[103]
Rutin	Orange juice	MOF/MoO_3_-Ppy NW/GCE (DPV)	0.50–2000/0.27 nM	[104]
AOC (as caffeic acid)	Wine, grape	C fiber UME (CV)	3–500/0.41 µM	[105]
AOC (as caffeic acid)	Wine	PEDOT/GCE (DPV)	7.5–75/1.48 µM	[106]
AOC (as Trolox)	Tea, plant extracts, fruit juice	Cu(II)-Neocuproine/Carrageenan-MWCNT/GCE (CA)	4.98–84.39/0.59 µM	[107]
AOC (DPPH)	Teas	LIGE (BIA/CA)	2–900/0.6 µM	[108]
TP (as catechol)	Mate tea	FeTsPc/SPE (CV)	2–120/0.6 µM	[109]

Modifiers: bex = banana pulp extract; CMC = carboxymethylcellulose; CuPTc = copper-phthalocyanine; F-GO = fluorinated graphene oxide; FeTsPc = iron tetrasulfonated phthalocyanine; g-C_3_N_4_NS = graphitic carbon nitrides nanosheets; GR = graphene; IL = ionic liquid; MAX = transition metal carbides; MIP = molecularly imprinted polymer; MNP = magnetic nanoparticles; MOF = metal–organic framework; MSi = mesoporous silica; MWCNT = multi-walled carbon nanotubes; NW = nanowires; OMC = ordered mesoporous carbon; Pal = palygorskite; PAM = polyacrylamide; PAN = polyacrylonitrile; PANI = polyaniline; PEDOT = poly(3,4-ethylenedioxythiophene); PmPD = poly(m-phenylenediamine); Ppy = polypyrrole; PST = polystyrene; TNT = highly ordered titanium dioxide nanotube arrays; ZIF = zeolitic imidazolate framework. Electrodes: GCE = glassy carbon; ITO = indium tin oxide; LIGE = laser-induced graphene; PGE = pencil graphite; SPE = screen-printed; UME = ultramicroelectrode. Techniques: BIA = batch-injection analysis; CA = chronoamperometry; CV = cyclic voltammetry; DPV = differential pulse voltammetry; PEC = photoelectrochemical detection; POT = potentiometry; SWV = square wave voltammetry.

**Table 4:** Main characteristics of the biosensors used for determining phenolic compounds.

Analyte	Sample	Sensor (Technique)	Linear Range/LOD	Ref.
Caffeic acid	Wine	Lacc/CS-Au NP/PEDOT-PSSLi/GCE (DPV)	2–90/1.9 µM	[111]
Gallic acid			2–18/1.7 µM	
Catechol	Fruit wines	Tyr/MWCNT/Ppy/GCE (CA)	1–60/5 µM	[112]
		PPO extract/bioPpy/GCE (CA)	1–70/2.1 µM	
AOC (as HQ)	Wine, syrup	Lacc/Au NP/GNPl/SPE (CA)	4–130/1.5 µM	[113]
TP (as catechol)	Olive oil	Lacc/CS-GM/AuE (CV)	10^−10^–100/10^−10^ µM	[114]
TP (as tyrosol)	Beer	Tyr/Au NP/SPE (CA)	2.5–50/1.8 µM	[115]

Modifiers: CS = chitosan; GM = galactomannan; GNPl = graphene nanoplatelets; Lacc = laccase; MWCNT = multi-walled carbon nanotubes; NP = nanoparticles; PEDOT = poly(3,4-ethylenedioxythiophene); PPO = polyphenol oxidase; Ppy = polypyrrole; PSSLi = poly(4-lithium styrenesulfonic acid); Tyr = tyrosinase. Electrodes: GCE = glassy carbon; SPE = screen-printed. Techniques: CA = chronoamperometry; CV = cyclic voltammetry. HQ = hydroquinone.

## Data Availability

Not applicable.

## References

[1] Cheung P.C.K., Mehta B.M. (2015). Handbook of Food Chemistry.

[2] Shahidi F., Ambigaipalan P. (2015). Phenolics and Polyphenolics in Foods, Beverages and Spices: Antioxidant Activity and Health Effects—A Review. J. Funct. Foods.

[3] Desimoni E., Brunetti B. (2013). Presenting Analytical Performances of Electrochemical Sensors. Some Suggestions. Electroanalysis.

[4] Derina K., Korotkova E., Barek J. (2020). Non-Enzymatic Electrochemical Approaches to Cholesterol Determination. J. Pharm. Biomed. Anal..

[5] Salemi Y., Hassanpoor S., Hassanpoor M. (2023). Electrochemical Determination of Phenylalanine in Biological, Food and Pharmaceutical Samples Using a MnO_2_/N-doped Reduced Graphene Oxide Nanocomposite. ChemistrySelect.

[6] Wong A., Materón E.M., Freitas T.A., Faria R.C., Gonçalves D., Del Pilar Taboada Sotomayor M. (2022). Voltammetric Sensing of Tryptophan in Dark Chocolate Bars, Skimmed Milk and Urine Samples in the Presence of Dopamine and Caffeine. J. Appl. Electrochem..

[7] Mousavi S.-F., Alimoradi M., Shirmardi A., Zare-Shahabadi V. (2020). Preparation, Characterization and Electrochemical Application of an Ag/Zeolite Nanocomposite: Application to Sub-Micromolar Quantitation of Tryptophan. J. Porous Mater..

[8] Prongmanee W., Alam I., Asanithi P. (2019). Hydroxyapatite/Graphene Oxide Composite for Electrochemical Detection of L-Tryptophan. J. Taiwan Inst. Chem. Eng..

[9] Ahmadi Direstani S., Dianat S. (2023). A Novel Bio-Electrochemical Sensor Based on a 1,4-Bis(Triphenylphosphonium)Butane)_3_ [SiW_11_O_39_Ni(H_2_O)]/P@ERGO Nanocomposite for the Selective Determination of l-Cysteine and l-Tryptophan. Mater. Adv..

[10] Garg S., Singh A., Parmar A.S., Rosy (2023). Boron Carbon Nitride-Assisted Electro-Functionalization of Screen-Printed Electrode for Tryptophan Sensing. ACS Appl. Nano Mater..

[11] Wang Y., Jiao J., Chu M., Jin Z., Liu Y., Song D., Yu T.-T., Yang G., Wang Y., Ma H. (2023). A Three-Dimensional Composite Film-Modified Electrode Based on Polyoxometalates and Ionic Liquid-Decorated Carbon Nanotubes for the Determination of L-Tyrosine in Food. Microchim. Acta.

[12] Jiang J., Wang X., Qi H., Han Y. (2024). Fabrication of Electrochemical Sensor Based on Nanocomposite of Zinc Oxide Nanoparticle-Molecularly Imprinted Polymer for Determination of Tyrosine in Food. J. Food Meas. Charact..

[13] Revenga-Parra M., Robledo S.N., Martínez-Periñán E., González-Quirós M.M., Colina A., Heras A., Pariente F., Lorenzo E. (2020). Direct Determination of Monosaccharides in Honey by Coupling a Sensitive New Schiff Base Ni Complex Electrochemical Sensor and Chemometric Tools. Sens. Actuators B Chem..

[14] Ayranci R., Demirkan B., Sen B., Şavk A., Ak M., Şen F. (2019). Use of the Monodisperse Pt/Ni@rGO Nanocomposite Synthesized by Ultrasonic Hydroxide Assisted Reduction Method in Electrochemical Nonenzymatic Glucose Detection. Mater. Sci. Eng. C.

[15] Yang S., Wang Y., Sheng Q. (2022). Heterostructural NiCo_2_O_4_ Nanocomposites for Nonenzymatic Electrochemical Glucose Sensing. Electroanalysis.

[16] He L., Su J., You T., Xiao S., Shen Y., Jiang P., He D. (2023). Mn Incorporation Boosted NiO Nanosheets as Highly Efficient Anode for Sensitive Glucose Detection in Beverage. LWT.

[17] Liu W., Zhao X., Dai Y., Qi Y. (2022). Study on the Oriented Self-Assembly of Cuprous Oxide Micro-Nano Cubes and Its Application as a Non-Enzymatic Glucose Sensor. Colloids Surf. B Biointerfaces.

[18] Tang L., Yan Y., He C., Zhou J., Yin J., Zhang Z., Tu L., Liu Z., Yang Q., He J. (2024). NiCo Layered Double Hydroxide Nanosheet Arrays Constructed via In-Situ Plasma Etching as Non-Enzymatic Electrochemical Sensor for Glucose in Food. Microchem. J..

[19] Kumar B., Sinha S.K. (2024). Growth of WO_3_ Nanostructure Deposited on TiO_2_ Nanotube Arrays for Electrochemical Enzyme-Free Glucose Sensor. J. Food Compos. Anal..

[20] Liu K., Wang X., Luo B., Wang C., Hou P., Dong H., Li A., Zhao C. (2022). Enzyme-Free Electrochemical Sensors for in Situ Quantification of Reducing Sugars Based on Carboxylated Graphene–Carboxylated Multiwalled Carbon Nanotubes–Gold Nanoparticle–Modified Electrode. Front. Plant Sci..

[21] Fernández I., González-Mora J.L., Lorenzo-Luis P., Villalonga R., Salazar-Carballo P.A. (2020). Nickel Oxide Nanoparticles-Modified Glassy Carbon Electrodes for Non-Enzymatic Determination of Total Sugars in Commercial Beverages. Microchem. J..

[22] Nasution M.A.F., Firmanti M.I., Riyanto H.G., Sanjaya A.R., Saepudin E., Ivandini T.A. (2023). Electrochemical and Computational Studies of Citrate-Modified β-cyclodextrin@Fe_3_O_4_ Nanocomposite as a Nonenzymatic Sensor for Cholesterol. Sens. Mater..

[23] Benešová L., Klouda J., Bláhová E., Nesměrák K., Kočovský P., Nádvorníková J., Barták P., Skopalová J., Schwarzová-Pecková K. (2022). Non-Enzymatic Electrochemical Determination of Cholesterol in Dairy Products on Boron-Doped Diamond Electrode. Food Chem..

[24] Abou-Elyazed A.S., Li S., Mohamed G.G., Li X., Meng J., EL-Sanafery S.S. (2023). Graphitic Carbon Nitride/MOFs Hybrid Composite as Highly Selective and Sensitive Electrodes for Calcium Ion Detection. Molecules.

[25] Yoon J.H., Park H.J., Park S.H., Lee K.G., Choi B.G. (2020). Electrochemical Characterization of Reduced Graphene Oxide as an Ion-to-Electron Transducer and Application of Screen-Printed All-Solid-State Potassium Ion Sensors. Carbon Lett..

[26] Özbek O. (2023). A Potentiometric Sensor for the Determination of Potassium in Different Baby Follow–on Milk, Water, Juice and Pharmaceutical Samples. J. Food Compos. Anal..

[27] Kul S.M., Chailapakul O., Sagdic O., Ozer T. (2024). A Smartphone-Based Sensor for Detection of Iron and Potassium in Food and Beverage Samples. Food Chem..

[28] Amayreh M., Hourani M.K. (2019). Determination of Iron in Spinach Using Linear Sweep Voltammetry at Iodine-Coated Platinum Rotating Disk Electrode. J. AOAC Int..

[29] Zoratti M., Frechero M.A., Centurión M.E. (2024). Voltammetric Sensor Based on Molybdenum-Vanadium-Lithium-Borate Glassy Matrix and Its Application for the Determination of Iron in Fortified Milk Powder. J. Anal. Chem..

[30] Kalayci S. (2021). A New Phosphate Selective Electrode and Its Application in Some Foods. Int. J. Electrochem. Sci..

[31] Tan Z., Wu W., Yin N., Jia M., Chen X., Bai Y., Wu H., Zhang Z., Li P. (2020). Determination of Selenium in Food and Environmental Samples Using a Gold Nanocages/Fluorinated Graphene Nanocomposite Modified Electrode. J. Food Compos. Anal..

[32] Wang H., Guo Y., Pan H. (2021). Determination of Selenium and Copper in Water and Food by Hierarchical Dendritic Nano-Gold Modified Glassy Carbon Electrodes. Analyst.

[33] Zhang L., Li S., O’Halloran K.P., Zhang Z., Ma H., Wang X., Tan L., Pang H. (2021). A Highly Sensitive Non-Enzymatic Ascorbic Acid Electrochemical Sensor Based on Polyoxometalate/Tris(2,2′-Bipyridine)Ruthenium (II)/Chitosan-Palladium Inorganic-Organic Self-Assembled Film. Colloids Surf. Physicochem. Eng. Asp..

[34] Duzmen S., Baytak A.K., Aslanoglu M. (2020). A Novel Voltammetric Platform Composed of Poly(Aminopyrazine), ZrO_2_ and CNTs for a Rapid, Sensitive and Selective Determination of Ascorbic Acid in Pharmaceuticals and Food Samples. Mater. Chem. Phys..

[35] López-Pastor J.-A., Martínez-Sánchez A., Aznar-Poveda J., García-Sánchez A.-J., García-Haro J., Aguayo E. (2020). Quick and Cost-Effective Estimation of Vitamin C in Multifruit Juices Using Voltammetric Methods. Sensors.

[36] De Faria L.V., Lisboa T.P., De Farias D.M., Araujo F.M., Machado M.M., De Sousa R.A., Matos M.A.C., Muñoz R.A.A., Matos R.C. (2020). Direct Analysis of Ascorbic Acid in Food Beverage Samples by Flow Injection Analysis Using Reduced Graphene Oxide Sensor. Food Chem..

[37] Vaschetti V.M., Viada B.N., Tamborelli A., Eimer G.A., Rivas G.A., Dalmasso P.R. (2022). Ultrasensitive Multiwall Carbon Nanotube-Mesoporous MCM-41 Hybrid-Based Platform for the Electrochemical Detection of Ascorbic Acid. Analyst.

[38] Bian L., Wang X., Liu D. (2024). Enhanced Detection of Vitamin C in Sports Beverages Using Electrochemical Sensors with Nano-Gold Particles. J. Food Meas. Charact..

[39] Zhang Y., Han M., Peng D., Qin H., Zheng H., Xiao J., Yang N. (2024). MOF-Derived High-Density Carbon Nanotubes “Tentacle” with Boosting Electrocatalytic Activity for Detecting Ascorbic Acid. Talanta.

[40] Čižmek L., Komorsky-Lovrić Š. (2020). Electrochemistry as a Screening Method in Determination of Carotenoids in Crustacean Samples Used in Everyday Diet. Food Chem..

[41] Jashari G., Muriqi S., Arbneshi T., Metelka R., Švancara I., Sýs M. (2021). A New Voltammetric Approach for the Determination of β-Carotene in Vegetables and Pharmaceutical Capsules Using a Gold Electrode. Talanta.

[42] Khan S., Wong A., Rychlik M., Sotomayor M.D.P.T. (2022). A Novel Synthesis of a Magnetic Porous Imprinted Polymer by Polyol Method Coupled with Electrochemical Biomimetic Sensor for the Detection of Folate in Food Samples. Chemosensors.

[43] Silva-Neto H.A., Barbeira P.J.S., Coltro W.K.T., Piccin E. (2024). 3D Printing of Electrochemical Cell for Voltammetric Detection and Photodegradation Monitoring of Folic Acid in Juice Samples. Food Chem..

[44] Xu Y., Gao X., Tao T., Ji L., Liu M., Zhang X., Xiao D. (2024). Sensitive Electrochemical Determination of Quercetin and Folic Acid with Cobalt Nanoparticle Functionalized Multi-Walled Carbon Nanotube. Microchim. Acta.

[45] Bandyopadhyay D., Nag S., Das D., Banerjee Roy R. (2024). Electrochemical Detection of Folic Acid in Food Extracts Using Molecularly Imprinted Polyacrylonitrile Imbued Graphite Electrode. Anal. Chim. Acta.

[46] Porada R., Fendrych K., Kochana J., Baś B. (2022). Simple and Reliable Determination of B Group Vitamins in Various Food Matrices with the Use of the Voltammetric Sensor Based on Ni-Zeolite/Carbon Black Nanocomposite. Food Control.

[47] Tesfaye G., Negash N., Tessema M. (2022). Sensitive and Selective Determination of Vitamin B_2_ in Non-Alcoholic Beverage and Milk Samples at Poly (Glutamic Acid)/Zinc Oxide Nanoparticles Modified Carbon Paste Electrode. BMC Chem..

[48] Thangphatthanarungruang J., Yakoh A., Laocharoensuk R., Chotsuwan C., Chailapakul O., Siangproh W. (2020). High-Efficient of Graphene Nanocomposite: Application to Rapidly Simultaneous Identification and Quantitation of Fat-Soluble Vitamins in Different Matric Samples. J. Electroanal. Chem..

[49] Avan A.A., Filik H. (2021). Simultaneous Determination of Fat-Soluble Vitamins by Using Modified Glassy Carbon Electrode. Russ. J. Electrochem..

[50] Martins E.C., Santana E.R., Spinelli A. (2023). Nitrogen and Sulfur Co-Doped Graphene Quantum Dot-Modified Electrode for Monitoring of Multivitamins in Energy Drinks. Talanta.

[51] Jesadabundit W., Chaiyo S., Siangproh W., Chailapakul O. (2020). Simple and Cost-Effective Electrochemical Approach for Monitoring of Vitamin K in Green Vegetables. ChemElectroChem.

[52] Muresan L.M. (2010). Zeolite-Modified Electrodes with Analytical Applications. Pure Appl. Chem..

[53] Costa-Rama E., Fernández-Abedul M.T. (2021). Paper-Based Screen-Printed Electrodes: A New Generation of Low-Cost Electroanalytical Platforms. Biosensors.

[54] Sharma V., Jayaprakash G.K. (2022). Fabrications of Electrochemical Sensors Based on Carbon Paste Electrode for Vitamin Detection in Real Samples: Review Paper. J. Electrochem. Sci. Eng..

[55] Liu Y., Li M., Li H., Wang G., Long Y., Li A., Yang B. (2019). In Situ Detection of Melatonin and Pyridoxine in Plants Using a CuO–Poly(l-Lysine)/Graphene-Based Electrochemical Sensor. ACS Sustain. Chem. Eng..

[56] Gao J., Li H., Li M., Wang G., Long Y., Li P., Li C., Yang B. (2021). Polydopamine/Graphene/MnO_2_ Composite-Based Electrochemical Sensor for in Situ Determination of Free Tryptophan in Plants. Anal. Chim. Acta.

[57] Shao T., Wang J., Noroozifar M., Kraatz H. (2023). Ferrocene-Peptide Conjugate for the Detection and Quantification of Ascorbic Acid. ChemElectroChem.

[58] Jiang C., Xie L., Yan F., Liang Z., Liang J., Huang K., Li H., Wang Y., Luo L., Li T. (2023). A Novel Electrochemical Aptasensor Based on Polyaniline and Gold Nanoparticles for Ultrasensitive and Selective Detection of Ascorbic Acid. Anal. Methods.

[59] Zhan T., Feng X.-Z., Cheng Y.-Y., Han G.-C., Chen Z., Kraatz H.-B. (2023). Electrochemical Sensor for Ultrasensitive Sensing of Biotin Based on Heme Conjugated with Gold Nanoparticles and Its Electrooxidation Mechanism. Food Chem..

[60] Arul P., Nandhini C., Huang S.-T., Gowthaman N.S.K., Huang C.-H. (2023). Tailoring of Peroxidase Mimetics Bifunctional Nanocomposite: Dual Mode Electro-Spectroscopic Screening of Cholesterol and Hydrogen Peroxide in Real Food Samples and Live Cells. Food Chem..

[61] Amor-Gutiérrez O., Costa-Rama E., Fernández-Abedul M.T. (2019). Sampling and Multiplexing in Lab-on-Paper Bioelectroanalytical Devices for Glucose Determination. Biosens. Bioelectron..

[62] Amor-Gutiérrez O., Costa-Rama E., Fernández-Abedul M.T. (2021). Fully Integrated Sampler and Dilutor in an Electrochemical Paper-Based Device for Glucose Sensing. Microchim. Acta.

[63] Pagkali V., Soulis D., Kokkinos C., Economou A. (2022). Fully Drawn Electrochemical Paper-Based Glucose Biosensors Fabricated by a High-Throughput Dual-Step Pen-on-Paper Approach with Commercial Writing Stationery. Sens. Actuators B Chem..

[64] Xia J., Zou B., Liu F., Wang P., Yan Y. (2022). Sensitive Glucose Biosensor Based on Cyclodextrin Modified Carbon Nanotubes for Detecting Glucose in Honey. J. Food Compos. Anal..

[65] Artigues M., Oh S., Gilabert-Porres J., Abellà J., Borrós S., Colominas S. (2019). Novel Grafted Electrochemical Interface for Covalent Glucose Oxidase Immobilization Using Reactive Pentafluorophenyl Methacrylate. Colloids Surf. B Biointerfaces.

[66] Artigues M., Gilabert-Porres J., Texidó R., Borrós S., Abellà J., Colominas S. (2021). Analytical Parameters of a Novel Glucose Biosensor Based on Grafted PFM as a Covalent Immobilization Technique. Sensors.

[67] Monkrathok J., Janphuang P., Suphachiaraphan S., Kampaengsri S., Kamkaew A., Chansaenpak K., Lisnund S., Blay V., Pinyou P. (2024). Enhancing Glucose Biosensing with Graphene Oxide and Ferrocene-Modified Linear Poly(Ethylenimine). Biosensors.

[68] Jadán Piedra F., Rojas C., Latorre Castro G.B., Maldonado Alvarado P. (2023). Selective Determination of Lysine in Mozzarella Cheese Using a Novel Potentiometric Biosensor. Food Biotechnol..

[69] Nohwal B., Chaudhary R., Kumar P., Pundir C.S. (2020). Fabrication and Application of an Amperometric Lysine Biosensor Based on Covalently Immobilized Lysine Oxidase Nanoparticles onto Au Electrode. Int. J. Biol. Macromol..

[70] Song Y., Yao J., Wang R., Wang C., Zhao Y., Wang L. (2021). A Photoelectrochemical Biosensor Based on SnO_2_ Nanoparticles for Phosphatidylcholine Detection in Soybean Oil. Anal. Methods.

[71] Yu D., Zou D., Li D., Wang X., Zhang X., Yu C., Wang L., Elfalleh W., Jiang L. (2019). Detection of Phosphatidylcholine Content in Crude Oil with Bio-Enzyme Screen-Printed Electrode. Food Anal. Methods.

[72] Bagal-Kestwal D.R., Chiang B.-H. (2019). Platinum Nanoparticle-Carbon Nanotubes Dispersed in Gum Arabic-Corn Flour Composite-Enzymes for an Electrochemical Sucrose Sensing in Commercial Juice. Ionics.

[73] David M., Florescu M., Bala C. (2020). Biosensors for Antioxidants Detection: Trends and Perspectives. Biosensors.

[74] Manikandan V.S., Sidhureddy B., Thiruppathi A.R., Chen A. (2019). Sensitive Electrochemical Detection of Caffeic Acid in Wine Based on Fluorine-Doped Graphene Oxide. Sensors.

[75] Tu X., Xie Y., Gao F., Ma X., Lin X., Huang X., Qu F., Ping L., Yu Y., Lu L. (2020). Self-Template Synthesis of Flower-like Hierarchical Graphene/Copper Oxide@copper(II) Metal-Organic Framework Composite for the Voltammetric Determination of Caffeic Acid. Microchim. Acta.

[76] Gao Y., Jin C., Zhang X., Li J., Wang F., Zhang Y. (2022). Determination of Caffeic Acid Using a Glassy Carbon Electrode Modified with Porous Carbon Material Obtained from Tetrapanax Papyriferus. Ionics.

[77] Magerusan L., Pogacean F., Pruneanu S. (2022). Eco-Friendly Synthesis of Sulphur-Doped Graphenes with Applicability in Caffeic Acid Electrochemical Assay. Bioelectrochemistry.

[78] Dos Santos D.E.F., Baumgarten L.G., Martins E.C., Dreyer J.P., Santana E.R., Winiarski J.P., Vieira I.C. (2024). A Sustainable Nanomaterial Based on Gold Nanoparticles and Graphene for Highly Sensitive Electrochemical Sensing of Caffeic Acid in Coffees. Food Anal. Methods.

[79] Yang Y., Li J., Wang Y., Liu Z., Xie Y., Zhao P., Hu X., Fei J. (2024). ZIF-Derived Porous Carbon Loaded Multicomponent Heterostructured Yolk@shell Nanospheres as an Ultrasensitive Electrochemical Sensing Platform for the Detection of Caffeic Acid in Food. Electrochim. Acta.

[80] Shabani E., Zappi D., Berisha L., Dini D., Antonelli M.L., Sadun C. (2020). Deep Eutectic Solvents (DES) as Green Extraction Media for Antioxidants Electrochemical Quantification in Extra-Virgin Olive Oils. Talanta.

[81] Sekar S., Huijun J., Liuzhu Z., Jin C., Lee S., Kim D.Y., Manikandan R. (2022). Copper Phthalocyanine Conjugated Graphitic Carbon Nitride Nanosheets as an Efficient Electrocatalyst for Simultaneous Detection of Natural Antioxidants. Electrochim. Acta.

[82] Montemerlo A.E., Azcarate S.M., Camiña J.M., Messina G. (2024). Chemometrically Assisted Differential Pulse Voltammetry for Simultaneous and Interference-Free Quantification of Gallic and Caffeic Acids. Anal. Methods.

[83] Della Pelle F., Rojas D., Scroccarello A., Del Carlo M., Ferraro G., Di Mattia C., Martuscelli M., Escarpa A., Compagnone D. (2019). High-Performance Carbon Black/Molybdenum Disulfide Nanohybrid Sensor for Cocoa Catechins Determination Using an Extraction-Free Approach. Sens. Actuators B Chem..

[84] Abd-Rabboh H.S.M., Amr A.E.-G.E., Naglah A.M., Almehizia A.A., Kamel A.H. (2021). Effective Screen-Printed Potentiometric Devices Modified with Carbon Nanotubes for the Detection of Chlorogenic Acid: Application to Food Quality Monitoring. RSC Adv..

[85] Gao Q., Zang Y., Zhang Y., Xie J., Li J., Gao J., Xue H. (2020). Composite Polymerized Molecular Imprinting Membrane-Based Electrochemical Sensor for Sensitive Determination of Curcumin by Using 4-Pentenoyl-Aminoacyl-Chitosan Oligosaccharide as Functional Monomer Oligomer. J. Electroanal. Chem..

[86] Li W., Jiang Z., Tan L., Wang S., Wang C., Zhang J., Zhou L., Zhang Q., Yuan C. (2020). Rapid Measurements of Curcumin from Complex Samples Coupled with Magnetic Biocompatibility Molecularly Imprinted Polymer Using Electrochemical Detection. J. Sep. Sci..

[87] Wang X., Tan W., Wang Y., Wu D., Kong Y. (2019). Electrosynthesis of Poly(m-Phenylenediamine) on the Nanocomposites of Palygorskite and Ionic Liquid for Electrocatalytic Sensing of Gallic Acid. Sens. Actuators B Chem..

[88] Yang T., Zhang Q., Chen T., Wu W., Tang X., Wang G., Feng J., Zhang W. (2020). Facile Potentiometric Sensing of Gallic Acid in Edible Plants Based on Molecularly Imprinted Polymer. J. Food Sci..

[89] Zhao H., Guo M., Li F., Zhou Y., Zhu G., Liu Y., Ran Q., Nie F., Dubovyk V. (2023). Fabrication of Gallic Acid Electrochemical Sensor Based on Interconnected Super-P Carbon Black@mesoporous Silica Nanocomposite Modified Glassy Carbon Electrode. J. Mater. Res. Technol..

[90] Magerusan L., Pogacean F., Rada S., Pruneanu S. (2022). Sulphur-Doped Graphene Based Sensor for Rapid and Efficient Gallic Acid Detection from Food Related Samples. J. Taiwan Inst. Chem. Eng..

[91] Rojas D., Della Pelle F., Del Carlo M., Fratini E., Escarpa A., Compagnone D. (2019). Nanohybrid Carbon Black-Molybdenum Disulfide Transducers for Preconcentration-Free Voltammetric Detection of the Olive Oil o-Diphenols Hydroxytyrosol and Oleuropein. Microchim. Acta.

[92] Şenocak A. (2020). Fast, Simple and Sensitive Determination of Coumaric Acid in Fruit Juice Samples by Magnetite Nanoparticles-zeolitic Imidazolate Framework Material. Electroanalysis.

[93] Saljooqi A., Shamspur T., Mostafavi A. (2020). Fe_3_O_4_@SiO_2_-PANI-Au Nanocomposite Prepared for Electrochemical Determination of Quercetin in Food Samples and Biological Fluids. Electroanalysis.

[94] Tesfaye G., Hailu T., Ele E., Negash N., Tessema M. (2021). Square Wave Voltammetric Determination of Quercetin in Wine and Fruit Juice Samples at Poly (Safranine O) Modified Glassy Carbon Electrode. Sens. Bio-Sens. Res..

[95] Liv L., Karakuş E. (2023). Signal-Enhanced Electrochemical Determination of Quercetin with Poly(Chromotrope Fb)-Modified Pencil Graphite Electrode in Vegetables and Fruits. ACS Omega.

[96] Sarikaya İ., Kaleoğlu E., Çakar S., Soykan C., Özacar M. (2023). An Enhanced Photosensitive Sensor Based on ITO/MWCNTs@Polymer Composite@BiVO_4_ for Quercetin Detection. Biosensors.

[97] Luo G., Deng Y., Zhu L., Liu J., Zhang B., Zhang Y., Sun W., Li G. (2020). Au-Co Nanoparticles-Embedded N-Doped Carbon Nanotube Hollow Polyhedron Modified Electrode for Electrochemical Determination of Quercetin. Microchim. Acta.

[98] Takahashi S., Muguruma H., Osakabe N., Inoue H., Ohsawa T. (2019). Electrochemical Determination with a Long-Length Carbon Nanotube Electrode of Quercetin Glucosides in Onion, Apple Peel, and Tartary Buckwheat. Food Chem..

[99] Huang S., Yang J., Li S., Qin Y., Mo Q., Chen L., Li X. (2021). Highly Sensitive Molecular Imprinted Voltammetric Sensor for Resveratrol Assay in Wine via Polyaniline/Gold Nanoparticles Signal Enhancement and Polyacrylamide Recognition. J. Electroanal. Chem..

[100] Zhang Q., Zhang C., Ying Y., Ping J. (2021). An Easy-Fabricated Ordered Mesoporous Carbon-Based Electrochemical Sensor for the Analysis of Trans-Resveratrol in Red Wines. Food Control.

[101] Yang X., Guo Q., Yang J., Chen S., Hu F., Hu Y., Lin H. (2021). Synergistic Effects of Layer-by-Layer Films for Highly Selective and Sensitive Electrochemical Detection of Trans-Resveratrol. Food Chem..

[102] Şenocak A., Sanko V., Tümay S.O., Orooji Y., Demirbas E., Yoon Y., Khataee A. (2022). Ultrasensitive Electrochemical Sensor for Detection of Rutin Antioxidant by Layered Ti_3_Al_0.5_Cu_0.5_C_2_ MAX Phase. Food Chem. Toxicol..

[103] Bakhnooh F., Arvand M. (2023). A Novel “Signal-off” Photoelectrochemical Sensing Platform for Selective Detection of Rutin Based on Cu_2_SnS_3_/TiO_2_ Heterojunction. J. Photochem. Photobiol. Chem..

[104] Wang Y., Chen Y., Zhou Y., Wang Y., Wu Y., Xie Y., Zhao P., Hu X., Fei J. (2024). Ultra-Sensitive Electrochemical Sensor Based on in Situ Grown Ultrafine HKUST-1 Nanoparticles @Graphite Nanosheets and Core–Shell Structured MoO_3_-Polypyrrole Nanowires for the Detection of Rutin in Orange Juice. Microchim. Acta.

[105] Gevaerd A., Silva B.M.D., Oliveira P.R.D., Marcolino Júnior L.H., Bergamini M.F. (2020). A Carbon Fiber Ultramicroelectrode as a Simple Tool to Direct Antioxidant Estimation Based on Caffeic Acid Oxidation. Anal. Methods.

[106] Fort C.I., Cobzac C.S.A., Turdean G.L. (2024). Conductive Polymer-Based Modified Electrode for Total Antioxidant Capacity Determination. Microchem. J..

[107] Şen F.B., Elmas E., Dilgin Y., Bener M., Apak R. (2024). Amperometric Sensor for Total Antioxidant Capacity Measurement Using Cu(II)-Neocuproine/Carrageenan-MWCNT/GCE. Microchem. J..

[108] Inoque N.I.G., Araújo D.A.G., De Lima D.M., Sousa R.M.F., Paixão T.R.L.C., Munoz R.A.A. (2024). Rapid Quantification of Antioxidant Capacity in Tea Using Laser-Induced Graphene Electrodes. ACS Food Sci. Technol..

[109] Martin C.S., Maximino M.D., Pereira M.S., Alessio P. (2019). Polyphenol Detection in Mate Tea Samples Using Iron Tetrasulfonated Phthalocyanine Modified Screen-Printed Electrode. IEEE Sens. J..

[110] Brainina K., Tarasov A., Khamzina E., Stozhko N., Vidrevich M. (2020). Contact Hybrid Potentiometric Method for On-Site and in Situ Estimation of the Antioxidant Activity of Fruits and Vegetables. Food Chem..

[111] Krzyczmonik P., Klisowska M., Leniart A., Ranoszek-Soliwoda K., Surmacki J., Beton-Mysur K., Brożek-Płuska B. (2023). The Composite Material of (PEDOT-Polystyrene Sulfonate)/Chitosan-AuNPS-Glutaraldehyde/as the Base to a Sensor with Laccase for the Determination of Polyphenols. Materials.

[112] Apetrei R.-M., Cârâc G., Bahrim G., Camurlu P. (2019). Utilization of Enzyme Extract Self-Encapsulated within Polypyrrole in Sensitive Detection of Catechol. Enzym. Microb. Technol..

[113] Zrinski I., Pungjunun K., Martinez S., Zavašnik J., Stanković D., Kalcher K., Mehmeti E. (2020). Evaluation of Phenolic Antioxidant Capacity in Beverages Based on Laccase Immobilized on Screen-Printed Carbon Electrode Modified with Graphene Nanoplatelets and Gold Nanoparticles. Microchem. J..

[114] Boubezari I., Bessueille F., Bonhomme A., Raimondi G., Zazoua A., Errachid A., Jaffrezic-Renault N. (2020). Laccase-Based Biosensor Encapsulated in a Galactomannan-Chitosan Composite for the Evaluation of Phenolic Compounds. Biosensors.

[115] Cerrato-Alvarez M., Bernalte E., Bernalte-García M.J., Pinilla-Gil E. (2019). Fast and Direct Amperometric Analysis of Polyphenols in Beers Using Tyrosinase-Modified Screen-Printed Gold Nanoparticles Biosensors. Talanta.

